# Impact of prematurity and nutrition on the developing gut microbiome and preterm infant growth

**DOI:** 10.1186/s40168-017-0377-0

**Published:** 2017-12-11

**Authors:** Alex Grier, Xing Qiu, Sanjukta Bandyopadhyay, Jeanne Holden-Wiltse, Haeja A. Kessler, Ann L. Gill, Brooke Hamilton, Heidie Huyck, Sara Misra, Thomas J. Mariani, Rita M. Ryan, Lori Scholer, Kristin M. Scheible, Yi-Horng Lee, Mary T. Caserta, Gloria S. Pryhuber, Steven R. Gill

**Affiliations:** 1 Genomics Research Center, University of Rochester School of Medicine and Dentistry, Rochester, NY USA; 2 Department of Biostatistics and Computational Biology, University of Rochester School of Medicine and Dentistry, Rochester, NY USA; 3 Department of Microbiology and Immunology, University of Rochester School of Medicine and Dentistry, 601 Elmwood Avenue, Rochester, NY 14642 USA; 4 Division of Neonatology, Department of Pediatrics, University of Rochester School of Medicine and Dentistry, Rochester, NY USA; 5 Division of Infectious Disease, Department of Pediatrics, University of Rochester School of Medicine and Dentistry, Rochester, NY USA; 6 Pediatric Molecular and Personalized Medicine Program, Department of Pediatrics, University of Rochester School of Medicine and Dentistry, Rochester, NY USA; 7 Department of Pediatrics, Medical University of South Carolina, Charleston, SC USA; 8 Division of Pediatric Surgery, Department of Surgery, Robert Wood Johnson University Hospital, New Brunswick, NJ USA; 9 Department of Biological Sciences, Vanderbilt University, Nashville, TN USA

**Keywords:** Preterm infants, Gut microbiota, Phase transition, Meconium, Nutrition, Infant growth

## Abstract

**Background:**

Identification of factors that influence the neonatal gut microbiome is urgently needed to guide clinical practices that support growth of healthy preterm infants. Here, we examined the influence of nutrition and common practices on the gut microbiota and growth in a cohort of preterm infants.

**Results:**

With weekly gut microbiota samples spanning postmenstrual age (PMA) 24 to 46 weeks, we developed two models to test associations between the microbiota, nutrition and growth: a categorical model with three successive microbiota phases (P1, P2, and P3) and a model with two periods (early and late PMA) defined by microbiota composition and PMA, respectively. The more significant associations with phase led us to use a phase-based framework for the majority of our analyses. Phase transitions were characterized by rapid shifts in the microbiota, with transition out of P1 occurring nearly simultaneously with the change from meconium to normal stool. The rate of phase progression was positively associated with gestational age at birth, and delayed transition to a P3 microbiota was associated with growth failure. We found distinct bacterial metabolic functions in P1–3 and significant associations between nutrition, microbiota phase, and infant growth.

**Conclusion:**

The phase**-**dependent impact of nutrition on infant growth along with phase**-**specific metabolic functions suggests a pioneering potential for improving growth outcomes by tailoring nutrient intake to microbiota phase.

**Electronic supplementary material:**

The online version of this article (10.1186/s40168-017-0377-0) contains supplementary material, which is available to authorized users.

## Background

Nutrition in early life is a critical factor in neonatal growth and long-term health. Managing nutritional intake in preterm infants is a significant clinical challenge, with optimal nutrition and feeding regimens not resolved despite extensive study [1]. Even with continuing improvements in preterm infant care, > 50% will be discharged with ongoing severe postnatal growth failure [2]. Due to the profound impact of postnatal growth failure on health over an entire lifespan, it is imperative that we understand the clinical and nutritional variables that contribute to a poor outcome.

Recent studies on metabolism and metabolic diseases suggest that the infant gut microbiome directly impacts growth and development of preterm infants [3–6]. Maturation of the gut microbiota in early life is linked to physiological development, with long-term influences on factors that affect infant health [7–9]. While colonization of the infant gut microbiota is thought to begin within days of birth, observations of microbiota in the placenta and amniotic fluid suggest that initial seeding of infants by pioneering colonizers occurs in utero [10–13]. Evidence in premature infants suggests that microbiota development is driven by host biology and associated with gestational age [5], but is also shaped by the restricted environment in the neonatal intensive care unit (NICU), infant nutrition, and common clinical practices in neonatal care [14–20]. We hypothesize that assembly and function of the preterm infants’ gut microbiota is associated with postnatal growth patterns and represents a yet unexplored personalized therapeutic potential for optimizing infant development.

Development of the premature infant gut microbiota has recently been shown to evolve in a patterned progression associated with postmenstrual age (PMA; gestational age at birth plus week of life), dominated by *Bacilli* at early PMA, followed by *Gammaproteobacteria* and then *Clostridia* [5]. In our study, we introduce two models to identify associations between the microbiota, nutritional intake, medication, and preterm infant growth: a categorical model based on three quantitatively defined “phases” (P1, P2, and P3) that corresponds to three states of the microbiota and a PMA-based model with the three composition-based phases replaced by two constant time periods identified as early (< 34 weeks PMA; *n* = 362 data points) and late (≥ 34 weeks PMA; *n* = 343 data points). Our analyses identified more significant associations with phase, which led us to use the phase-based framework to explore potential functional relationships between the preterm microbiota, nutrition, and growth.

We first defined the phases and phase transition points in longitudinal gut microbiota samples from two cohorts of preterm and full-term infants from the multicenter Prematurity and Respiratory Outcomes Program (PROP) and Respiratory Pathogens Research Center (RPRC) at the University of Rochester School of Medicine. The distinguishing composition and putative functional capacity of each phase was assessed, along with the properties of transitions between phases. Our data suggest phase-specific microbiota functions and demonstrate the effect of nutritional intake and clinical factors on phase and period-specific microbiota development. Furthermore, our results indicate significant associations between nutritional intake, the phase of the microbiota, and preterm infant growth. Finally, we demonstrate that transition out of phase 1 (P1) occurs simultaneously with transition from meconium to normal postnatal stool, a milestone that can be unambiguously identified at the bedside.

Overall, our results illustrate an ecological framework for the preterm infant gut microbiome and represent a significant first step in tailoring nutrient intake according to microbiota phase. Thus, our study will inform and contribute to establishing much needed clinical criteria for managing microbiota-based nutrient intake and care that supports optimal infant growth and development.

## Results

### Overview of preterm infant cohort

Our study examined associations between preterm infant PMA, growth, nutrition, clinical factors, and gut microbiota development in a cohort of 95 preterm and 2 full-term infants from PROP and 23 full-term infants from RPRC at the University of Rochester School of Medicine. A total of 719 rectal swab samples were collected weekly from the PROP preterm infants while in the NICU, spanning PMA from 24 to 46 weeks with a good representation across gestational ages. A total of 2 rectal swabs were collected from the 2 PROP full-term infants, and 46 rectal swabs from the 23 RPRC full-term infants: one near birth (≤ 20 day of life [DOL]) and—for the RPRC subjects only—a second at 1 month of age (20 < DOL ≤ 50) (Table 1). The longitudinal analyses included 719 samples from preterm and 48 samples from full-term infants. Relevant available age metrics were gestational age at birth, day of life (DOL), and PMA. To select the age metric for our analyses, we used functional data analysis to fit nutrition and growth variables, as well as the abundance of operational taxonomic units (OTUs) in the microbiota, first using PMA and then DOL as the age variable [21]. The overall fitting variance using PMA was lower for OTU abundance and, for most metrics of growth and nutrition, consistent with previous findings that the temporal dynamics of the preterm infant gut microbiota correspond better to PMA than to DOL [5]. Consequently, we used gestational age at birth and PMA for our analyses.

**Table 1:** Demographic and clinical variables

Variables (*N* = 120)	Values (mean ± SD or *N*)
Gestational age at birth (weeks, preterm/full-term, mean ± SD)	28.83 ± 3.37/39.72 ± 1
Gestational age at birth (23–25/26–27/28–29/30–31/32–33/34–35 weeks/full-term, *N*)	25/22/11/11/18/8/25
Birth Weight (Kg, preterm/full-term, mean ± SD)	1.29 ± 0.57/3.6 ± 0.45
Sex (male/female, *N*)	59/61
Race (Caucasian/AA/Asian/Other, *N*)^a^	76/29/1/14
Ethnicity (Hispanic or Latino Y/N/unnkown, *N*)	13/103/4
Delivery method (C-section/vaginal, *N*)	60/60
Necrotizing enterocolitis diagnosis (medical, surgical, *N*)^b^	11
Received antimicrobials (any, ≥ 7 days, *N*)^c^	82 / 63
Received diuretics (any, *N*)^c^	54
Received postnatal corticosteroids (any, *N*)^c^	22
Received proton pump inhibitors (any, *N*)^c^	7
Received H_2_ receptor antagonist (any, *N*)^c^	13
Received motility agents (any, *N*)^c^	24

^a^Other race includes those unknown

^b^Necrotizing enterocolitis diagnosis (medical, surgical) diagnosis for one baby is unknown because of early withdrawal from study

^c^ Medication received within 1 week prior to microbiome sample collection. Sample collection occurred approximately weekly throughout the majority of the hospitalization as clinically permitted. If the interval between samples was greater than 7 days apart, then short portions of the hospital stay are not covered by this analysis. Therefore, subjects who had the respective medication, but not within 1 week prior to sample collection, are not counted in this summary table

To select nutritional and clinical variables for our study, we first used an initial linear mixed effect regression that associates gestational age at birth, PMA, one main covariate (see Additional file 1: Table S1 for the full list of covariates), and its interaction with PMA, with microbiota taxa abundance. We found that mode of delivery was not significantly associated with microbiota composition after controlling for age, which is similar to recent studies where mode of delivery was not associated with the influence of breast milk in preterm infants or with stool composition in full-term infants [22, 23]. Overall, PMA has the strongest impact on microbiota composition, followed by the ratio of enteral calories, total calories normalized by body weight, proportion of dietary lipids, antimicrobial usage, proportion of dietary protein, and diuretics usage (Additional file 1: Table S1). Corticosteroids, H_2_ receptor antagonists, and motility agent usage also have limited associations with some taxa. Based on these results, we applied a full linear mixed-effect regression model to identify the associations between microbiota phase, gestational age at birth, nutrient intake, and medication.

### Evaluation of fecal microbiota sampling methods

To develop a sampling protocol that yields highly reproducible and representative fecal microbiota profiles, we first compared fecal microbiota obtained from matched stools or meconium and rectal swabs from five infants. Both sampling methods identify similar composition of operational taxonomic units (OTUs) and alpha diversity or evenness of observed OTUs within each subject (*p* values > 0.1 between stool-meconium and rectal swabs) and greater diversity between subjects (Additional file 2: Figures S1 and S2). Results from differential abundance testing on a per taxon basis between the three groups (stool, meconium, and rectal swab) are not significant. Differential abundance testing between two groups (stool-meconium and rectal swab) identified one adjusted *p* value < 0.1 (0.088) for the *Clostridiales*. In comparison, evaluation of fecal microbiota collected as matched stool and swab samples in other studies demonstrated by composition and diversity metrics that microbiota from both samples is nearly identical [24, 25]. Based on similarity of alpha diversity and the ability of clinical NICU staff to collect and store samples directly from infants at specific times without cross contamination from infant diapers and skin, we selected rectal swabs as the preferred method for sampling gut microbiota.

### Three phases of the preterm infant gut microbiota

**Fig. 1:**
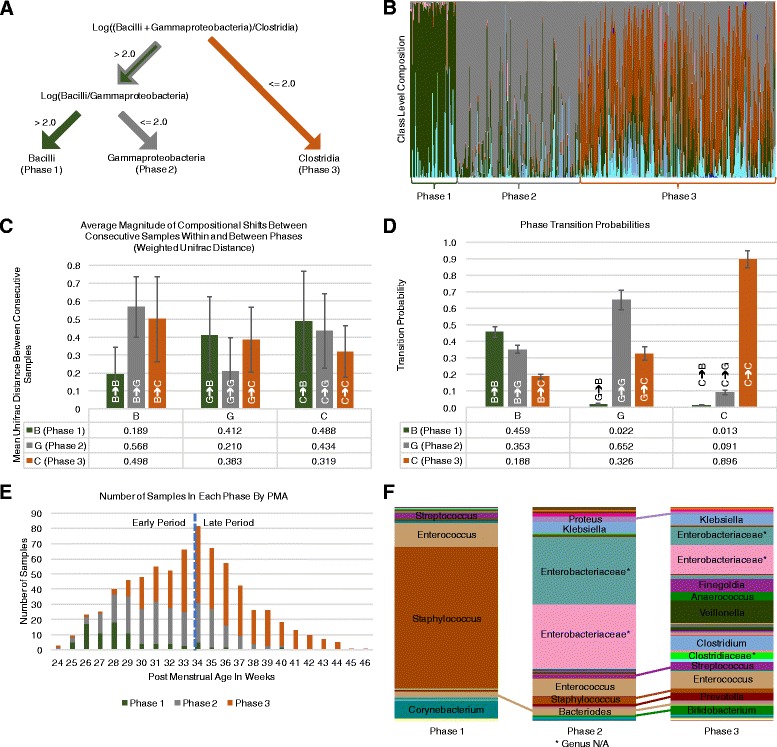
Overview of the preterm infant gut microbiota phases and properties. **a** The decision tree for classifying a microbiota sample into one of the three phases. **b** A composition bar chart with each sample grouped by phases 1–3 (P1–P3) from left to right. Green, gray, orange, and blue represent *Bacilli*, *Gammaproteobacteria*, *Clostridia*, and *Bacteroidia*, respectively. **c** Bar charts representing the average weighted UniFrac distance between consecutive samples of each individual infant. The bars are grouped into three major categories from left to right according to the initial phase of the consecutive samples being assessed. Each bar within a category corresponds to the phase of the second consecutive sample. The height of the bar indicates the average dissimilarity between consecutive samples of the corresponding phases, with exact values included in the table below the graph. **d** Bar charts indicating the transition probability between consecutive samples within an individual. The groupings and bars within each group indicate the phases of the first and second sample of a pair of consecutive samples, respectively, and are ordered as described in **c**. Transition probabilities are included in the table below the graph. **e** The distribution of samples over corrected gestational age in weeks. The dashed line separates the samples into early (< 34 weeks PMA) and late period (≥ 34 weeks PMA), based on functional variance of microbiota composition across all 81 individuals. **f** Bar charts showing the average composition of the samples in each phase at the genus level, with prominent genera labeled. For two *Enterobacteriacaea* and one *Clostridiacaeae*, the genus could not be determined and the family is indicated instead (See Additional file 2: Comment on Figure 3F). Lines connecting segments between phases indicate that the segment represents the same genus in each bar. A complete list of the genera represented here and their relative abundances can be found in Additional file 4: Table S3

Characterization of microbiota from all subjects and time points spanning PMA from 24 to 46 weeks identified *Bacilli*, *Gammaproteobacteria*, and *Clostridia* as by far the most abundant taxa, with relative abundances of 41.75, 23.0, and 22.5% respectively, accounting for 87.0% of the total observed abundance (Fig. 1a). The next most abundant classes are *Actinobacteria* and *Bacteroidia*, which account for just 6.5 and 5.1% of total observed abundance, respectively. To characterize the apparent developmental phases of the premature infant gut microbiome, we used threshold values for the log ratio of the three predominant bacterial classes—*Bacilli*, *Gammaproteobacteria*, and *Clostridia*—to construct a decision tree that permits objective assignment of individual microbiota samples to one of the three phases based on their composition (Fig. 1a and Methods). We used criteria that distinguished the phases based on their association with prematurity and lower PMA and on the relative dominance of *Bacilli*, *Gammaproteobacteria*, and *Clostridia* in P1, P2, and P3 respectively. The composition of individual samples assigned to each phase at the class level using these criteria is shown in Fig. 1b. The categorical structure of the model, which assumes relative stability within a phase and abrupt shifts in composition between phases, was validated by examining the week-to-week changes of the microbiota within each subject. Quantitative changes in the weekly microbiota samples were determined using weighted UniFrac distance to measure the dissimilarity between consecutive samples. Averaged over all subjects, consecutive samples of the same phase show substantially less dissimilarity week-to-week than consecutive samples of the differing phases (i.e., samples before and after a phase transition; Fig. 1c). Testing the median dissimilarity revealed that it is significantly higher when the phase changed between consecutive samples than when it remained the same (*p* < 0.0001), suggesting discrete periods of community restructuring corresponding to phase transition.

We quantified the pattern of progression of the gut microbiota with respect to the order of phase transition events using the sequence of phases observed in the consecutive samples from each individual infant to compute the transition probabilities between the phases. For each phase, the probability that the subsequent sample from the same individual will be in the same phase is higher than the probability of transitioning to a different phase (Fig. 1d). Transition from one phase to the next consecutive phase is more likely than the transition from a higher phase to a lower phase (e.g., P2 to P1) or from P1 to P3 directly. Accordingly, we found a strong relationship between PMA and microbiota phases (Fig. 1e). For this preterm cohort, 70% of all P1 samples were observed at PMA of 29 weeks or less; 84% of all P2 samples were observed from 28 weeks to 36 weeks PMA; and 78% of all P3 samples at 33 weeks or later. Eighty six percent of all samples from 37 weeks PMA and later were in P3, suggesting that preterm infant gut physiology and developmental stage influences the microbiota following birth.

For a point of comparison with our phase-based clustering, we performed Dirichlet multinomial mixture (DMM) modeling, using the composition of each sample at the class level as input. The optimal model fit was achieved with four Dirichlet components (Additional file 2: Figures S3 and S4A–B and Additional file 3: Table S2). DMM component three corresponds to our P1 cluster, component two corresponds to P2, and components one and four correspond to P3. A majority of all samples (89.9%) were classified as representing the Dirichlet component matching the phase of the sample. DMM components one and four within the P3 cluster correspond to more and less mature sub-types. DMM component four was the more mature sub-type, with the average sample occurring 2 weeks after the average component one sample, and exhibited the canonical characteristics of P3 (high *Clostridia*, high diversity) with little or no recognizable characteristics of P2. DMM component one was the less mature sub-type, with samples exhibiting the distinguishing characteristics of P3 while retaining to some extent features of P2 (relatively high *Gammaproteobacteria*). The high concordance observed between DMM components and phases provides statistically grounded support for our heuristic model (See Additional file 3: Table S2 for details).

### Variance and abundance of taxa across three microbiota phases

Taxonomic analysis of all samples identified 16 phyla, 38 classes, 73 orders, 158 families, and 383 genera. Compositional differences across the phases at all taxonomic levels were characterized and pairwise comparisons were made between phases. The average composition of the samples in each phase at the genus level is shown in Fig. 1f, which represents the abundance of genera relative to bar size. The most significantly differentially abundant taxa between P2 and P3 were a variety of *Clostridiales* elevated in P3, including the genera *Veillonella*, *Finegoldia*, *Clostridium*, and *Anaerococcus*. The most significant differences between P1 and P3 were observed among *Staphylococcaceae* which were elevated in P1, and among *Clostridiales* genera *Finegoldia* and *Veillonella* which were elevated in the P3. A complete list of differentially abundant taxa can be found in Additional files 4 and 5: Tables S3 and S4A–C.

### Functional capacity of microbiota phases

**Fig. 2:**
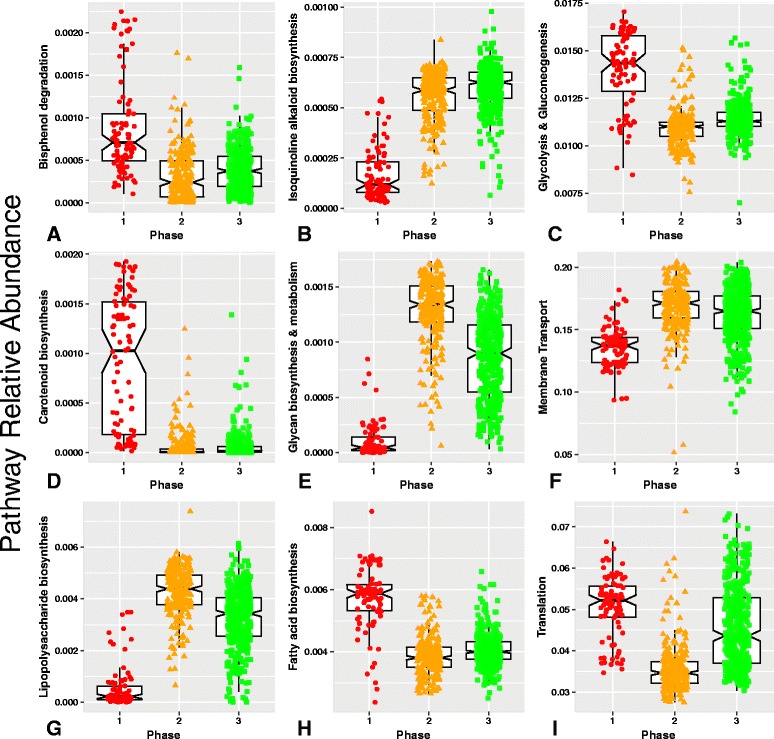
**a**–**i** Functional capacity of microbiota phases. The functional capacity of the microbiota present in each sample was inferred using PICRUSt (Phylogenetic Investigation of Communities by Reconstruction of Unobserved States) [63]. Each gray panel corresponds to one function and each point within a gray panel represents one sample. The samples are stratified by phase along the *x* axis, with red circles corresponding to P1, orange triangles corresponding to P2, and green squares corresponding to P3 samples. The sample position on the *y* axis indicates the relative abundance of the specified KEGG pathway, calculated as the fraction of times functional components of that pathway occurs across all organisms in the sample, with the contribution of each organism weighted by its relative abundance. Within each phase, samples are plotted on top of a box plot, which is centered on the median, with notches indicating an approximately 95% confidence interval, boxes indicating the boundaries of the first and third quartiles, and whiskers extending to the largest and smallest values no further than 1.5*(inter-quartile range) from the boxes. Points beyond the whiskers are outliers. If the notches of two boxes within the same gray panel do not overlap on the *y* axis, there is strong evidence that the true medians differ [69]. Functional pathways that are differentially enriched among the three phases include those that contribute to the degradation of phthalates on NICU medical devices (bisphenol degradation), protection against oxidative stress (carotenoid biosynthesis), microbiota driven increases in lipopolysaccharide (LPS) concentrations (lipopolysaccharide biosynthesis), short-chain fatty acids (fatty acid biosynthesis), isoquinoloine alkaloid biosynthesis, glycolysis and gluconeogenesis, glycan biosynthesis and metabolism, membrane transport and translation

The inferred functional capacity of the microbiota was compared between the three phases, revealing differences potentially relevant to nutrient processing and microbiota-derived metabolites that contribute to establishment and maintenance of gut mucosal homeostasis (Fig. 2). P1 exhibited enrichment for bisphenol A (BPA) degradation and carotenoid synthesis pathways with BPA being an environmental contaminant frequently found in preterm infants due to repeated exposure to plastics in medical devices [26–28] and carotenoids conferring protection of gut microbiota against oxidative stress [29, 30]. Additional pathways were found to be significantly differentially abundant when comparisons were made between phases, including an increased capacity for synthesis of isoquinoline alkaloids, glycan and lipopolysaccharide (LPS) in P2 and P3. Protein translation, fatty acid biosynthesis and glycolysis and gluconeogenesis were increased in P1. A complete list of differentially abundant putative functions can be found in Additional file 6: Table S5.

### Effect of microbiota phase on infant growth, nutrient intake, and medication

These observations prompted us to explore the potential relationship between microbiota phase, parenteral and enteral nutrient intake, medication and infant growth. Using linear mixed-effect regression models that account for subject-specific variation, with microbiota phase, gestational age at birth, nutrient intake, and medication as explanatory variables, we assessed the association of weight Z-score (standard deviation score (Z-score) of infant’s weight based on weight percentiles of a reference population matched for prematurity and sex; used as the dependent variable) and these covariates. Significant associations with weight Z-score include gestational age at birth, the phase of the microbiota, the ratio of major macronutrients, the proportion of calories administered enterally, the receipt of motility agents, antibiotics, diuretics, corticosteroids, and several interaction terms between medication/nutrition and the phase of the microbiota (Table 2). The significance with respect to weight Z-score of interaction terms between relative lipid and protein intake and the phase of the microbiota indicate that the observed association between these macronutrients and growth depends on the composition of the gut microbiota and differs between microbiota phases.

**Table 2:** Associations of weight Z-score with microbiome phase, nutrition, and clinical covariates

Significant covariates	*p* value	Beta value
Ratio of proteins to total calories (g/cal) * phase 2	0.0071	10.95
Ratio of lipids to total calories (g/cal) * phase 2	0.0437	5.658
Phase 2	0.0003	− 0.7766
Calories/kilogram past week	< 0.0001	− 0.626
Proportion of calories enteral	< 0.0001	− 0.5566
Corticosteroids past week * phase 1	0.0035	0.3669
Diuretics past week * phase 1	0.0002	0.3393
Diuretics past week * phase 2	< 0.0001	0.3306
Motility agents past week * phase 2	0.0003	− 0.3144
Diuretics past week	< 0.0001	− 0.2266
Antibiotics past week	< 0.0001	− 0.2176
Motility agents past week	0.0087	0.1522
Gestational age at birth	0.0165	0.059

Multiple regression associations of weight Z-score (as the outcome variable) with microbiome phase, nutrition, and other clinical covariates, as well as the interactions between phases and clinical covariates (interaction terms denoted with an asterisk). *P* values indicate the significance of each association while beta values indicate the direction and magnitude of the relationship between weight Z-score and the covariates

**Fig. 3:**
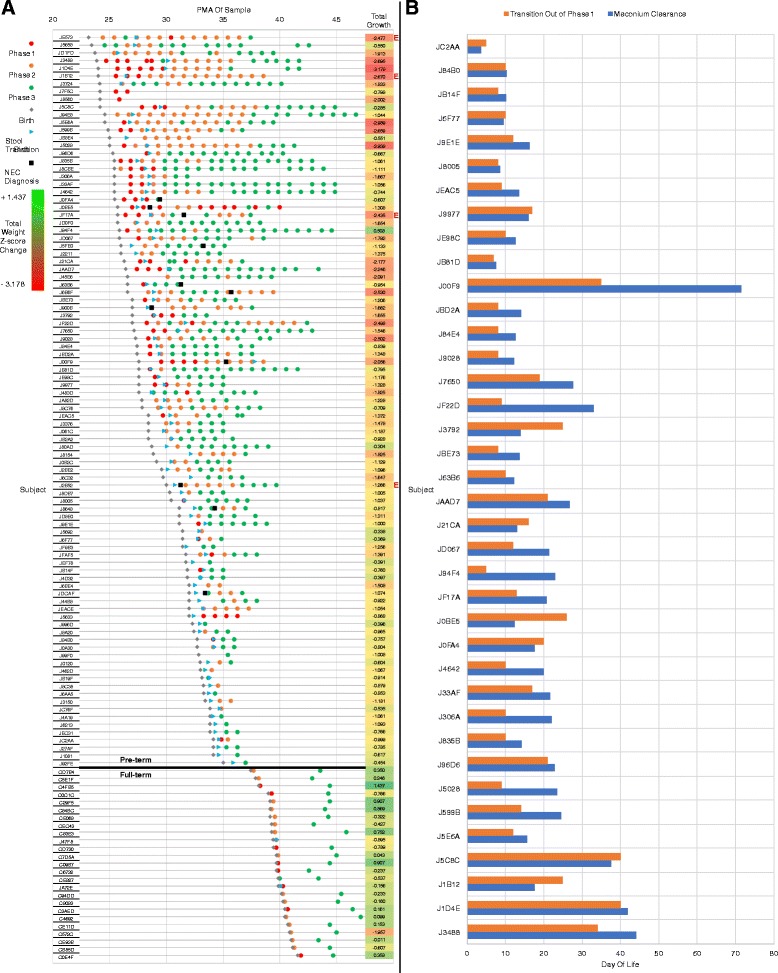
Temporal distribution of gut microbiota phases, change in infant weight and meconium clearance. **a** All rectal samples from 95 preterm and 25 full-term infants are plotted against post menstrual age, stratified by subject and sorted by gestational age at birth. Samples for preterm infants include those collected weekly from birth through discharge. Samples for full-term infants include the first sample after birth (collected at ≤ 20 DOL) and a second sample, collected ≤ 50 DOL. Microbiota phases (P1, red circle; P2, orange circle; P3, green circle), birth (gray diamond), stool transition (blue arrowhead), and NEC diagnosis (black square) at discharge are also indicated. Change in weight Z-score from birth to discharge, and elemental feeding requirements (maroon E) at discharge for preterm infants are indicated in the right margin. The lowest to greatest change in weight Z-score from birth to discharge spans the spectrum from red to green. In all infants, except for J94F4, the total weight change in weight Z-score from birth to discharge was negative. Weight Z-score changes in full-term infants were both positive and negative, and negative changes tended to be smaller than those observed in preterms. **b** Day of life of stool transition and phase transition for 38 preterm subjects in phase one (P1) at the time of their first microbiota sample. The relationship between day of life (DOL) for the initial transition out of P1 and from meconium to normal infant stool was modeled by linear regression. These results demonstrate a highly significant association between the transition out of P1 and from meconium to normal infant stool that is independent of PMA or prematurity, suggesting that the P1 and meconium microbiota are closely associated

The longitudinal patterns of rectal microbiota phase transitions for 95 preterm and 25 full-term subjects are shown in Fig. 3a relative to gestational age at birth. Growth of the subjects is shown as change in weight Z-score from birth to NICU discharge for preterms and birth to 1 month for full terms. Comparison of preterm infants in P1 (*N* = 42; mean birth GA (gestational age) = 27.43 weeks) with those in P2 or P3 (*N* = 55; mean birth GA = 30.29 weeks) at the time of their first microbiota sample showed significant difference (*p* < 0.0001) in mean birth GA between these two groups. Furthermore, the most premature subjects (< 29 weeks birth GA) were significantly more likely to be in P1 than the full-term subjects at their first sample (61.8 vs 32.0%, *p* = 0.025). The change in weight Z-score is associated with length of time in each phase, with the lowest change (at the red end of the spectrum in Fig. 3a) in subjects (i.e., JE573, J5028, J1B12) who remain in phase 1 or 2 for prolonged periods. The largest negative change in weight Z-scores was associated with delays in transition to a P3 gut microbiota (*p* = 0.0023). Delayed achievement of P3 was also associated with prematurity, with full-term subjects reaching P3 by 1 month of age much more frequently than preterm subjects (100 vs 53.4%, *p* = 0.0001). Similarly, greater PMA-adjusted growth by discharge (preterms) or 1 month (full terms) was observed in the full-term subjects than in the preterm subjects (mean change in weight Z-score − 0.033 vs − 1.269, *p* ≈ 0.0). Eleven infants were treated for necrotizing enterocolitis (NEC) and two of these died of the disease. In those who survived, NEC was frequently followed by more than 2 weeks in P2 (J6B6F, J900B, J00F9, J2B52, and J8648). One infant who required a jejunal ostomy remained in P1 for an extended period of time (J0BE5). Thus, prolonged periods in P1 and P2 may represent the effects of lengthy antibiotic treatment and/or lack of enteral nutrition. Although the number of cases is insufficient for statistical assessment, our data suggest an association between delayed transition to P3 and a long-standing feeding intolerance in the NICU that results in administration of elemental amino acid-based formula (maroon ‘E’ in the right-hand margin of Fig. 3a).

### Effect of nutrition and medication on microbiota taxa in each phase

We next examined the effects of nutrient intake and medications on the microbiota within each phase. Changes in taxa abundance would suggest adaptation of the microbiota in response to factors that the preterm infant encounters while in the NICU and may affect development of a mature, functional gut microbiota. Using a multivariate mixed-effects regression model that accounts for subject-specific variations, we assessed changes in taxa abundance at each phase, with PMA, gestational age at birth, total calories per kilogram in the past week, proportion of enteral calories in the past week, ratio of lipids, carbohydrates and protein in the past week, antibiotics and corticosteroid received during the past week, and additional medications as covariates. We found phase-specific changes in the microbiota significantly associated with the ratio of lipids, proteins, or carbohydrates in nutrition (total enteral and parenteral), with the dominant effect of all three nutrients in P3. At the phylum level, *Actinobacteria* and *Proteobacteria* are significantly associated with lipid intake, *Firmicutes* with protein and *Actinobacteria*, *Proteobacteria*, and *Firmicutes* with carbohydrates. Abundance of *Bifidobacterium*, an *Actinobacterium* most commonly linked with development and maintenance of the healthy infant gut microbiota [31, 32], is significantly associated with lipid and protein intake in P3, with increased *Bifidobacterium* abundance associated with increased lipid in the diet and decreased abundance with greater amounts of protein. Among the commonly used NICU medications, increased abundance of *Bifidobacterium* was significantly associated with use of corticosteroids and H_2_ receptor antagonists in the past week in P3. A complete list of nutrition and medication variables significantly associated with changes in microbiota taxa in all the three phases can be found in in Additional file 7: Table S6.

### Early and late periods of the preterm infant microbiome

To demonstrate the utility of modeling the microbiome as three compositionally defined phases, we compared gut microbiota development using two constant time periods based solely on PMA. Specifically, all preterm longitudinal samples (*n* = 705) were divided into two groups or periods of equal functional variance based on fitted microbiota taxa abundance, an early period (< 34 weeks PMA; *n* = 362) and a late period (≥ 34 weeks PMA; *n* = 343) (Fig. 1e and Additional file 2: Figure S5). This separation into early and late periods was used as an unbiased point of comparison to assess the utility of the phase-based approach relative to a purely temporal approach in the context of a nutrition-medication-microbiota-growth model. Using linear mixed-effects regression models as described for the phase-based nutrition analysis (Methods-Model A) with the weight Z-score as an outcome variable, we identified more significant associations in the phase based model than the period based model. The complete results of the period-based models can be found in Additional file 8: Table S7. Overall, our results demonstrate that the phase-based model of gut microbiota development in the preterm infant provides a more robust explanation of the data than the period-based model.

### Potential associations of nutrition and medications with phase

After identifying the microbiota phase as a significant factor in infant growth, we sought to identify potential associations of nutrition and medications with phase by including them as explanatory variables and microbiota phases P1 and P2 as the outcome (with P3 as the baseline phase) in a multivariate mixed-effects logistic regression model. We completed separate analyses to identify associations during the early and late periods with nutrition and medications. Postmenstrual age, nutrient ratios, and the proportion of calories from enteral feeding are significantly associated with the phase of the microbiota in both periods. A higher proportion of nutritional lipids is consistently positively associated with the infant gut microbiota being in P2 and negatively associated with P1, while a higher proportion of proteins is positively associated with a P1 microbiota at an earlier PMA, negatively associated with being in P1 at later PMA, and negatively associated with being in P2 irrespective of PMA. Antibiotics are positively associated with a P2 gut microbiota, significant in the later PMA period (*p* = 0.0015), and nearly significant in the earlier period (*p* = 0.0778). The variables used in these analyses, as well as their *p* values and beta estimates, are provided in Table 3A–B and Additional file 9: Table S8A–B.

**Table 3:** Significant results of mixed effects logistic regression for nutrition and medication

Covariates	Phase 1 *p* value	Phase 2 *p* value	Phase 1 beta value	Phase 2 beta value
A^a^				
Ratio of lipids to total calories (g/cal)	0.1913	0.0002	− 57.74	119.8
Ratio of proteins to total calories (g/cal)	0.0092	0.0053	110.1	− 70.12
Proportion of calories enteral	0.8786	< 0.0001	− 0.2702	− 6.039
Diuretics past week	0.007	0.1053	− 1.457	0.5927
PMA (week)	0.032	0.1674	− 0.3831	0.1403
B^b^				
Ratio of proteins to total calories (g/cal)	0.9455	0.0259	− 84.96	− 98.05
Proportion of calories enteral	0.7124	0.0017	15.29	− 6.428
Antibiotics past week	0.8903	0.0014	9.91	1.903
PMA (week)	0.3467	0.0011	− 7.108	− 0.4875

^a^Results of mixed-effects logistic regression analysis between nutrition/medication and microbiome phases during the EARLY period (< 34 weeks PMA). Phases 1 and 2 are considered as binary outcome variables (yes/no) and are analyzed separately. Beta values are the estimated regression coefficients and *p* values are computed from the likelihood ratio tests. For clarity, only significant associations are reported in this table. Full results are reported in Additional file 9: Table S8A

^b^Results of mixed-effects logistic regression analysis between nutrition/medication and microbiome phases during the LATE period (≥ 34 weeks PMA). Phases 1 and 2 are considered as binary outcome variables (yes/no) and are analyzed separately. Beta values are the estimated regression coefficients and *p* values are computed from the likelihood ratio tests. For clarity, only significant associations are reported in this table. Full results are reported in Additional file 9: Table S8B

### Association between the meconium microbiota and transition out of phase 1

In addition to nutrition and other external factors that may influence the phase of the gut microbiome, we sought to identify dynamic aspects of host biology that correspond to phase transition. Emerging evidence suggests that the initial newborn infant gut microbiota is partially acquired by maternal transmission from the amniotic fluid and placenta before birth [10–13]. In utero, the fetus swallows large quantities of amniotic fluid that is colonized with bacteria in those mothers who deliver prematurely [11, 33]. Genera in common between amniotic fluid and the meconium, the earliest fecal material passed by infants, suggests that pioneer colonizers of the infant gut are from this maternal source. In addition to amniotic fluid that has been consumed, meconium is formed from sloughed off gastrointestinal epithelial cells which are generated as debris during periods of rapid digestive tract development and convolution of the intestinal epithelial surface. It has been established that in preterm infants, passage of meconium as stool is both delayed and prolonged and is observed well beyond the first stool, with final clearance occurring up to several weeks after birth [34].

To identify potential associations between the presence of meconium and P1 of the microbiota, we examined the relationship between clearance of the meconium from the stool and the initial transition of the microbiota out of P1 (Fig. 3b). Two infants remained in P1 (J7F5C and J8560), but did not survive beyond the first weeks in the NICU. Two infants cleared their meconium by discharge but their last microbiota sample was still in P1 (J0BE5 and J5633). The remaining 38 infants that were observed to be in P1 at their first rectal sample were included in a linear regression model using gestational age at birth and the DOL of their last P1 sample before their initial transition to another phase as explanatory variables, with the DOL of meconium clearance as the dependent variable. This model explained approximately half of the variation in the day of life of stool transition from meconium to normal infant stool (R-square = 0.51). Phase transition was found to be highly significant in this model (*p* < 0.0001), while gestational age at birth and the intercept did not exhibit significant associations (*p* values = 0.35 and 0.23, respectively), indicating that the time of stool transition was not associated with prematurity or PMA once microbiota phase transition was controlled for. On average, the last P1 sample before the initial phase transition occurred 4.7 days before stool transition was observed (Fig. 3b). To assess the similarity between the meconium and P1 microbiota samples, we first categorized all 721 samples as meconium or not, depending on whether the sample was collected from an infant that had not transitioned to normal stool, and as P1 or not, according to the decision tree. A majority of meconium samples were in P1 (59.8%) and P1 samples in meconium (64.4%) (Additional file 2: Figure S6A). We next used linear regression analysis to identify the taxa significantly associated with meconium and then again to identify those associated with P1 (Additional file 2: Figure S6B and Additional file 10: Table S9). The taxa that differ significantly between meconium and non-meconium samples are nearly identical to the taxa that differ significantly between P1 and P2-P3. These results demonstrate a highly significant association between the transition out of P1 and transition between meconium and normal infant stool, and that P1 and meconium share similar microbiota.

## Discussion

Development of the early life gut microbiome is a critical factor in neonatal survival and long-term health [5, 6, 35–38]. In this study, we examined the effects of nutrition and clinical practices in the NICU on development of the preterm gut microbiome and neonatal growth. The developing microbiota of preterm infants is dominated by three classes of bacteria, whose relative proportions are temporally defined [5]. In most infants, *Bacilli* initially dominate, followed by *Gammaproteobacteria*, and ultimate convergence to a state dominated by *Clostridia* by approximately 37 weeks PMA. We demonstrate that this process can be understood as a series of three ordered phases, with relative stability being maintained for a period within each phase and transitions between phases characterized by rapid, dramatic shifts in the composition of the microbial community. The phases and transition points between them can be defined quantitatively, allowing individual microbiota samples to be unambiguously categorized. Analyses of the putative functional capacity of the phases revealed marked differences and suggest significant roles in host metabolism and gastrointestinal development. While progression through the phases depends largely on PMA, we identified significant associations with nutritional factors, diuretics, and antibiotics. Notably, the initial transition out of P1 is significantly associated with the observed transition from meconium to normal stool. These results suggest that the gut microbiota of premature infants and its temporal dynamics may be best understood through a phase-based paradigm. We have demonstrated the utility of this paradigm by applying it to our examination of the relationships between the gut microbiota, nutrition, and growth.

The gut microbiota of preterm infants at birth is less diverse than in full-term infants and at a greater risk for dysbiosis due to physiological and immune immaturity and postnatal influences that disrupt developmental succession of the microbiota as they mature [23, 37, 39, 40]. Factors that influence microbiota development include prolonged hospitalization, postnatal medications, and formula feeding [16, 23, 37, 39, 41]. In this study, preterm infants were fed specialized premature base formulas or breast milk, which were then fortified with composition and volume guided by daily infant growth rates and clinical evaluation. All premature infants were supplemented with some liquid or powder formula fortification in order to target their higher macronutrient, phosphorous, and calcium targets set by current pediatric guidelines. It would be interesting to compare formula supplemented to exclusive maternal breastmilk intake, though this theoretical control group’s intake would place infants at unacceptable risk for growth failure. The composition and volume of formulas and volume of breast milk was monitored for each infant. Nutritional intake for each infant was calculated as the ratio of lipid, protein, and carbohydrate, total caloric intake, and proportion of enteral calories, normalized by body weight, and received the week prior to fecal sampling for microbiota analysis. We then evaluated the impact of these nutrients on successive phases of the microbiota in relationship to PMA and infant growth. Adjustment of enteral and parenteral intake of these nutrients, along with total calorie intake and medications, were associated with infant growth.

The succession of gut microbiota in our preterm cohorts revealed a low level of initial diversity in P1, which is dominated by facultative anaerobes, followed by increasing diversity and abundance of obligate anaerobes and a shift to fermentation based metabolism in P3. Consistent with other studies, we determined that this programmed, non-random developmental succession of microbiota is largely determined by PMA [5, 6]. What drives this patterned succession toward a homeostatic relationship between the infant and colonizing microbiota is not known, but likely involves complex interactions between the mucosal immune system as well as metabolic interactions within the gut microbial community and the surrounding tissue and microenvironment, which remain dynamic during periods of gastrointestinal development in early life. Antimicrobial peptides (AMPs) produced by Paneth cells (PCs) in the epithelium of the small intestine establish a feedback loop between the host and commensal bacteria that is essential for intestinal homeostasis and microbiota colonization [39]. Although the number of immune-competent PCs are significantly higher after 37 weeks of gestation when compared to preterm infants, the number of immune-competent PCs are higher in infants with GA above 29 weeks compared to infants with GA under 29 weeks [42]. This period around PMA 29 weeks corresponds to the PMA ~ 30 weeks where we observed the transition from P2 to P3 in our preterm cohorts, suggesting that PC AMPs are one factor that modulates the shift toward a community dominated by obligate anaerobes. *Streptococcus* and *Veillonella* in P3, which frequently co-occur and interact metabolically in microbial communities, through the production of lactic acid by *Streptococcus* which is used as a carbon source by *Veillonella* [43]. Similar metabolic interactions that contribute to succession and homeostasis will likely be identified through metabolic profiling of communities within each phase [6].

Phase-specific changes in the microbiota and infant growth were significantly associated with the ratio of lipids, proteins, and carbohydrates, and total caloric intake. Increased abundance of *Actinobacteria* and *Proteobacteria* was significantly associated with lipid intake, Firmicutes with protein, and *Actinobacteria*, *Proteobacteria* and *Firmicutes* with carbohydrates (Additional file 7: Table S6). A greater abundance of *Staphylococcus*, *Clostridium*, and *Enterococcus* as a result of an increased ratio of carbohydrates and total caloric intake in P3 reflects a potential change in the energy balance and increased growth due to a greater abundance of genes involved in lipid and carbohydrate metabolism and production of butyrate in these Firmicutes [44, 45]. While the increased ability of the microbiota to extract nutrients from the food consumed by the host may have a direct benefit for the preterm infant in early life, emerging data suggests the potential for a strong impact on disease programming and obesity in later life [46–48]. Importantly, the effect of nutrition on growth was dependent on the microbiota phase during which individual nutrients were administered. These findings are consistent with the inferred functional differences between the phases and suggest that diet and treatment can be optimized based on microbiota phase. A higher proportion of protein is significantly positively associated in early life with a P1 microbiota. Increased protein and lipids during P1 are strongly associated with a higher growth rate. In contrast, a higher proportion of protein is significantly negatively associated with a P2 microbiota, with increased proportions of total nutrition lipids and protein in P2 strongly associated with higher weight Z-score. Relative to P1 and P3, P2 is associated with lower weight and growth at a given time.

Significant associations with phase succession were identified with exposure to broad-spectrum antibiotics, gut motility agents, corticosteroids for treatment of bronchopulmonary dysplasia (BPD), histamine-2 receptor (H2)-blockers and proton pump inhibitors (PPI) that reduce gastric acidity and gastroesophageal reflux [41, 49, 50]. Previous studies evaluating use of antibiotics, H2-blockers and PPI in preterm infants identified a relationship between their use and development of necrotizing enterocolitis (NEC) [5, 51]. Treatment with H2-blockers has been shown to favor the proliferation of *Proteobacteria* over *Firmicutes* in fecal microbiota, which is also associated with development of NEC [41]. Eleven preterm infants with NEC were included in our study, but were not analyzed as an independent group, and therefore, we cannot directly associate phase with changes in microbiota and NEC. However, our analysis of phase succession demonstrated that treatment of our preterm infants with H2-blockers or PPI was associated with an increase in *Proteobacteria*, *Actinobacteria*, and *Bacteroidetes* in P2 (Additional file 7: Table S6). Exposure to antibiotics in P2-3 and P3 resulted in a decrease in *Firmicutes* and increase in *Proteobacteria*, respectively. Furthermore, the occurrence of NEC relative to phase transition suggests an association of NEC with microbiota reverse transitions from P2 to P1 (subject J0BE5) and P3 to P2 (JF17A and J6B6F) and delayed transition to P3 (J00F9, J900B, and J2B52) (Fig. 3a).

While microbiota phase transition presents an opportunity to optimize postnatal growth, weekly assessment of an individual infant’s gut microbiome to target nutritional therapy is not yet feasible. However, the association of meconium clearance with transition out of P1 suggests use of clearance as a cost-free bedside tool to assess the consequences and therapeutic potential of transition from P1 to P2 in preterm infants. Implementing a diet with increased proportions of lipids and proteins in P1 may enhance infant growth and promote transition to P2. A subsequent increase in the proportion of proteins after transition to P2, as indicated by clearance of meconium, could maximize growth and promote progression to P3, and could be followed by adjustment of overall enteral calories for optimal growth. Further investigation into clinical surrogates of the microbial transition to P3 would provide additional benefit to bedside care and assessment of nutrition on long-term infant development. Broadly, healthy growth and rapid gut microbiota development (transition through the three phases) occur in parallel and are hindered by prematurity. Further investigation of associations between phase progression and dietary macronutrients and common medications may suggest potential avenues for microbiotic-focused care aimed at optimizing growth and mitigating certain pathologies associated with prematurity.

The clearance of meconium and transition out of P1 is not always stable, with the microbiota of some infants reverting back to a meconium state or P1 after the initial transition to P2 (Fig. 3a). Furthermore, clearance of meconium occurs in P2 of some (e.g., J5028 in Fig. 3a) or whose first sample was in P2 (i.e., J94E8). Expansion of meconium clearance over P2 is also shown in Additional file 2: Figure S6, where a significant number of P1 samples were not identified as meconium. This may be due to our reliance on a clinical observation of meconium clearance to more solid fecal material. Another likely source is the dynamic environment of the preterm gut and changes in microbiota-gut epithelium interactions during rapid development [52]. In addition to the expected observation of delayed and prolonged meconium clearance due to hypomotility consistent with the immature preterm gastrointestinal tract, it may be that the continued accumulation of sloughed off epithelial cells during the period of rapid intestinal growth and convolution known to occur during the developmental period corresponding to the third trimester results in a mixture of normal stool and meconium.

Given that functional properties of the microbiome, metabolism, and host physiology are likely of paramount significance to our observations, additional experimental approaches to identify underlying microbiome mechanisms at each phase would be of substantial value. Additionally, as this was not a case-control study, our ability to rigorously assess the relationship between nutrition, the microbiota, and growth was limited. Significant associations identified between different nutrients, the phase of the microbiota, and growth depended upon the variation in nutritional intake that occurred as a matter of course, independent of our study. Controlling these factors in a systematic way would likely be highly informative, but would be difficult to achieve given the risks to the study population. We also acknowledge a study limitation with the proportion of breast milk received by each infant, in that we were unable to collect precise measurements of breastmilk volumes due to variability from feed to feed in breastmilk availability. We recommend that future studies control or measure this quantity more precisely. Finally, additional metrics of host gut physiology and immune and metabolic development can be incorporated into future studies as they may provide insights into the factors driving phase progression as well as the potential impact of phase transition on the newborn’s growth and development.

## Conclusion

To our knowledge, this is the first study to demonstrate an association between gut microbiota phase, nutritional intake, and growth of preterm infants. We first developed a discrete three phase-based model based upon quantitative categorical classification of the preterm gut microbiota, characterized the composition and putative functional capacity of the three microbiota phases, and described the properties of phase transition. We determined that transition from meconium to normal infant stool is associated with transition of the microbiota out of P1. Second, we identified significant associations between phase-specific gut microbiota functions, growth, nutritional intake, and medication. Third, in both the phase- and period-based models, the abundance of several dominant infant gut microbiota taxa (e.g., *Bifidobacterium*) [37, 53] were significantly affected by gestational age at birth, PMA at sampling, total calories and proportions of macronutrients consumed at the week of sampling, and multiple clinical variables. Collectively, this work lays the foundation for additional studies to determine causality leading to personalized microbiome medicine of preterm infants and new clinical guidelines with nutritional and medication recommendations based on infant growth and gut microbiota development.

## Methods

### Clinical methods

All study procedures were approved by the University of Rochester School of Medicine Internal Review Board (IRB) (Protocol # 37933). Infants included in the study were from the multicenter Prematurity and Respiratory Outcomes Program (PROP) and the Respiratory Pathogens Research Center (RPRC) at the University of Rochester School of Medicine and were cared for in a single-center Newborn Intensive Care Unit (NICU). Clinical care in terms of type and duration of antibiotic treatment, corticosteroids, diuretics, motility agents, and H_2_ receptor agonists as well as the timing and volume of feeds was at the discretion of treating physicians. Rectal swabs were used to collect fecal material from consented infants from 24 PMA until discharge and again at 6 months and 1 year for preterms and birth and 1 month for full terms. Each sample was collected by inserting a sterile Copan flocked nylon swab (Copan Diagnostics, Murrieta, CA) moistened with normal saline beyond the sphincters into the rectum and then twirled. Each sample was immediately placed into sterile buffered saline and stored at 4 °C for no more than 4 h. Samples were processed daily, which involved extraction of the fecal material from the swab in a sterile environment and immediately frozen at − 80 °C until DNA extraction. All sampling swabs, plasticware, buffers, and reagents used for sample collection and extraction of nucleic acids were sterile and UV-irradiated to insure no contamination from sources outside of the infant and sample.

### Derived medication and nutrition variables

For all medications considered, binary variables were derived for each sample that indicate whether or not a given medication was administered in the week (7 days) prior to sample collection. Weight Z-score was computed as a proxy for growth. First, weight percentile was computed as the percentage of weight measures of a population of the same sex and age that fall below the observed weight value. We applied Cole’s LMS method as used by CDC and WHO [54]. The standard growth chart is based on sex-matched premature infant population weight data collected by Fenton and Kim [55, 56]. Weight Z-scores were computed based on the corresponding weight percentiles. Four variables associated with each sample were derived for nutritional intake: total calories per kilogram in the week prior to sample collection, ratio of lipids or proteins in the week prior to sample collection, and the ratio of total calories in the week prior to sample collection that were consumed enterally (as opposed to parenterally). These values were computed based on detailed daily feeding records and the available nutrition facts for all formulas, supplements, and total parenteral nutrient preparations used in the NICU. Total calories per kilogram in the past week is the sum of total calories per kilogram per day for the 7 days prior to sampling. The proportion of enteral calories computed as the ratio of (grams of lipids/protein per kilogram) divided by (total calories per kilogram) for each day, summed over the 7 days prior to sampling. “Enteral calorie ratio past week” was computed as the total calories per kilogram consumed enterally in the week prior to sampling divided by the total calories per kilogram consumed (enterally and parenterally) in the same period.

### Genomic DNA extraction

Total genomic DNA was extracted with a modified method using the QIAGEN Fecal DNA kit and FastPrep mechanical lysis (MPBio, Solon, OH). 16S ribosomal RNA (rRNA) was amplified with Phusion High-Fidelity polymerase (Thermo Scientific, Waltham, MA) and dual indexed primers specific to the V3-V4 hypervariable regions (319F: 5′ ACTCCTACGGGAGGCAGCAG 3′; 806R: 3′ ACTCCTACGGGAGGCAGCAG 5′) [57]. Amplicons were pooled and paired-end sequenced on an Illumina MiSeq (Illumina, San Diego, CA) in the University of Rochester Genomics Research Center. Each sequencing run included (1) positive controls consisting of a 1:5 mixture of *Staphylococcus aureus*, *Lactococcus lactis*, *Porphyromonas gingivalis*, *Streptococcus mutans*, and *Escherichia coli* and (2) negative controls consisting of sterile saline.

### 16S rRNA sequence processing

Raw data from the Illumina MiSeq was first converted into FASTQ format 2 × 300 paired-end sequence files using the bcl2fastq program, version 1.8.4, provided by Illumina. Format conversion was performed without de-multiplexing and the EAMMS algorithm was disabled. All other settings were default. Sequence processing and microbial composition analysis were performed with the Quantitative Insights into Microbial Ecology (QIIME) software package [58], version 1.9. Reads were multiplexed using a configuration described previously [57]. Briefly, for both reads in a pair, the first 12 bases were a barcode, which was followed by a primer, then a heterogeneity spacer, and then the target 16S rRNA sequence. Using a custom Python script, the barcodes from each read pair were removed, concatenated together, and stored in a separate file. Read pairs were assembled using fastq-join from the ea.-utils package, requiring at least 40 bases of overlap and allowing a maximum of 10% mismatched bases. Read pairs that could not be assembled were discarded. The concatenated barcode sequences were prepended to the corresponding assembled reads, and the resulting sequences were converted from FASTQ to FASTA and QUAL files for QIIME analysis. Barcodes, forward primer, spacer, and reverse primer sequences were removed during de-multiplexing. Reads containing more than four mismatches to the known primer sequences or more than three mismatches to all barcode sequences were excluded from subsequent processing and analysis. Assembled reads were truncated at the beginning of the first 30 base window with a mean Phred quality score of less than 20 or at the first ambiguous base, whichever came first. Resulting sequences shorter than 300 bases or containing a homopolymer longer than six bases were discarded. Operational taxonomic units (OTU) were picked using the reference-based USEARCH (version 5.2) [59] pipeline in QIIME, using the May 2013 release of the GreenGenes 99% OTU database as a closed reference [60, 61]. An indexed word length of 128 and otherwise default parameters were used with USEARCH. Chimera detection was performed de novo with UCHIME, using default parameters [59]. OTU clusters with less than four sequences were removed, and representative sequences used to make taxonomic assignments for each cluster were selected on the basis of abundance. The RDP Naïve Bayesian Classifier was used for taxonomic classification with the GreenGenes reference database, using a minimum confidence threshold of .85 and otherwise default parameters [62]. Phylogenetic investigation of communities by reconstruction of unobserved states (PICRUSt) [63] was used with the provided pre-processed KEGG Orthologs database to infer the putative functional capacities of these communities.

### 16S rRNA microbiota data pre-processing

To ensure the quality of statistical analysis, microbiome samples with < 12,000 total reads were excluded from the subsequent data analyses. Microbiota abundance data were summarized at six different levels (level 2: PHYLUM–level 7: SPECIES). For characterization of the microbiota phases and within phase abundance analyses, raw relative abundance values were used. For beta diversity calculations, normalization by rarefaction at a depth of 12,000 reads was performed. For longitudinal abundance analyses, at each taxonomic level we excluded OTU units (taxa) with equal or more than 98% of exactly zero reads among the 705 samples. In total, 140 genera and 198 species are used for these statistical analyses. The abundance data were log2 transformed (log_2_(*x* + 1)) following normalization by cumulative sum scaling [64].

### Description of decision tree logic to define microbiota phases

Drawing on the microbial dysbiosis index described by Gevers et al. [65], the first step in the decision tree is to compute and evaluate the log of (total abundance of the classes increased in prematurity (*Bacilli* + *Gammaproteobacteria*)) over (total abundance of the class decreased in prematurity (*Clostridia*)). If this value is less than or equal to two, the gut microbiota is defined as being in phase 3. If the result of the first step in the tree is greater than two, a second step is taken where we compute and evaluate the log of (total abundance of the class increased in extreme prematurity (*Bacilli*)) over (total abundance of the class decreased in extreme prematurity (*Gammaproteobacteria*)). If the resulting value is less than or equal to two, the gut microbiota is defined as being in phase two; otherwise, it is defined as being in phase one (P1). In the event that the ratio is non-computable because *Clostridia* is entirely absent and the P1|P2 branch is taken, or the P1|P2 branch is taken and *Gammaproteobacteria* is absent, the microbiota is defined as being in P1 or the P1|P2 branch is taken and *Bacilli* is absent, the microbiota is defined as being in P2. If two of the three classes are absent, the microbiota is defined as being in the phase characterized by the class that is present. No samples were entirely devoid of all three classes, but such a case could not be resolved within this framework. Dirichlet multinomial mixture (DMM) modeling for comparative purposes was performed using the Dirichlet multinomial R package, which is based on Holmes et al. [66]. Class-level composition was used, and per sample normalization was performed by converting relative abundances to counts summing to 12,000 (the minimum read threshold for inclusion in analysis). The dmn function was used with default parameters and an arbitrary seed value of 11; count data was fit to one through ten Dirichlet components, and model fit was estimated using the Laplace metric.

### Functional capacity of microbiota phases

The functional capacity of the microbiota present in each sample was inferred using PICRUSt (Phylogenetic Investigation of Communities by Reconstruction of Unobserved States) [63], which reconstructs the functional composition of a microbial community sample using 16S rRNA phylogeny and a database of annotated reference genomes. For each functional pathway from the Kyoto Encyclopedia of Genes and Genomes (KEGG) that was putatively identified, comparisons were made between the phases using LEfSe, which identifies features that are statistically differentially abundant among biological classes (in this case phases) and then performs comparative tests between pairs of biological classes to identify where these features are significantly enriched or diminished.

### Comparing taxonomic composition, functional capacity, and week-to-week dissimilarity between phases

Analysis of variance of taxa abundance at all taxonomic levels across the three phases of the microbiota was conducted using a Kruskal-Wallis test, and the results are summarized in Additional file 4: Table S3. Differential abundance of taxa between each pair of two phases was assessed at each taxonomic level using the metagenomicsSeq zero-inflated Gaussian test [64], and the results are summarized in Additional file 5: Tables S4A–C. Testing for differential functional capacity between the phases was performed using LEfSe [67] with per-sample normalization to 1 M total counts, minimum effect size of 2.0, alpha of 0.1, an all-against-all strategy, and otherwise default parameters. The results are summarized in Additional file 6: Table S5. An exploratory test of the equality of the median of the week-to-week differences of samples within individual subjects between the cases where the phase remains the same and the cases where the phase changes was performed using the Wilcoxon rank-sum test. The *p* value reported for this test is approximate due to the paired nature of beta-diversity and the presence of repeated measures from the same subjects.

### Transition from meconium to solid stool

The point of stool transition from meconium to normal as described in the text was determined from nurses’ records subjectively characterizing diaper contents when they were changed. These records were available as free text and each entry was time stamped, with one entry for every time a diaper was changed. Stool transition was defined as the first such record without the word meconium that was followed by no more than two records containing the word meconium. To assess the associations between day of life (DOL) of stool transition and day of life of initial transition out of phase one, a simple linear regression model was used with DOL of transition out of phase 1, gestational age at birth as covariates, and DOL of stool transition as the outcome. A similar regression model was used to assess the association between growth and time to reach phase 3. The DOL of the first phase 3 sample observed for each subject and their gestational age at birth were used as covariates, and the total change in weight Z-score from birth to discharge was used as the outcome variable. This model included only the 81 subjects who reached phase three prior to discharge.

### Determination of early and late time periods

We applied functional principal component analysis to the microbiota abundance data [21]. The estimated temporal abundance function of taxon *v* and subject *I*, ${\widehat{x}}_{i,v}(t)$, was represented by a linear combination of eigen-functions as follows:

$$ {\widehat{x}}_{i,v}(t)={\widehat{\mu}}_v(t)+\sum \limits_{k=1}^{K_v}{c}_{ik,v}{\xi}_{k,v}(t). $$

Here, ${\widehat{\mu}}_v(t)$ is the estimated mean curve for the *v*th taxon, *ξ*_*k*, *v*_(*t*) is the *k*th eigen-function for this taxon, *K*_*v*_ is the number of top eigen-functions needed to explain ≥ 99% of total functional variation, and *c*_*ik*, *v*_ are the linear coefficients. On average, it takes 2.93 functional principal components to explain ≥ 99% of total variation at the species level. We calculated the total functional variance based on the fitted microbiota abundance at the species level. More specifically, we computed the pointwise variance function for each species from the smoothed temporal curves of abundance at the species level, then took the summation over all species used in this study

$$ {V}_v(t)=\frac{1}{N-1}\sum \limits_{i=1}^N{\left({\widehat{x}}_{i,v}(t)-{\overline{x}}_{\cdot, v}(t)\right)}^2,\kern0.5em \overline{V}(t):= \frac{1}{M}\sum \limits_{v=1}^M{V}_v(t). $$

Here, ${\overline{x}}_{\cdot, v}(t)$ represents the sample mean abundance function calculated from all subjects. $\overline{V}(t)$ represents the overall temporal variance at the species level. The maximum of $\overline{V}(t)$ occurred at PMA = 34 weeks (rounded to integers), which is illustrated in Additional file 2: Figure S5. Based on this cutoff, we define the EARLY period of PMA to be (0,34) and the LATE period to be [34,∞). The EARLY interval has 362 data points; the LATE interval has 343 data points.

#### Association between clinical variables and microbiota abundance in each phase

Within each phase independently, association testing between all taxa and clinical and nutritional factors of interest was performed by regressing the relative abundance of each taxon on these covariates: gestational age at birth, post menstrual age, total calories per kilogram in the past week, ratio of lipids in the past week, ratio of proteins in the past week, ratio of carbohydrates in the past week, proportion of total calories received enterally in the past week, whether antibiotics were received in the past week, whether diuretics were received in the past week, whether corticosteroids were received in the past week, whether motility agents were received in the past week, whether proton pump inhibitors were received in the past week, and whether H_2_ receptor antagonists were received in the past week. This was done using the MaAsLin algorithm [68] with subject as a random variable, without model selection, and with otherwise default parameters. The results are summarized in Additional file 7: Table S6.

### Association between nutrition/medication and growth

We performed linear mixed-effect regression analysis similar to the above model on both early and late periods (Model A) and three phases (Model B) to test the association between the nutrition/medication factors (as covariates) and weight Z-score as a proxy for growth (as the response variables). We included gaBirth (gestational age at birth) and PMA in the model to control for their possible confounding effects. More specifically, the following two linear mixed-effects regressions were performed.

Model A:

$$ {\mathrm{Weight}}_i\left({t}_j\right)={\mathrm{Period}}_i\left({t}_j\right){\beta}_{\mathrm{period}}+\sum \limits_{k=1}^K{\mathrm{NutriMed}}_{i,k}\left({t}_j\right){\beta}_k+\mathrm{Interactions}+{\alpha}_i+{\epsilon}_{ij}. $$

Model B:

$$ {\mathrm{Weight}}_i\left({t}_j\right)={\mathrm{Phase}}_i\left({t}_j\right){\beta}_{\mathrm{phase}}+\sum \limits_{k=1}^K{\mathrm{NutriMed}}_{i,k}\left({t}_j\right){\beta}_k+\mathrm{Interactions}+{\alpha}_i+{\epsilon}_{ij}. $$

Here, NutriMed_(*i*,*k*)_ (*t*_*j*_) is the *k*th clinical covariate for the *i*th subject measured at the *j*th time point. *β*_*k*_ is the corresponding linear coefficient (fixed effect); *α*_*i*_ is a random-effect term that quantifies the within-subject dependence; and *ϵ*_*ij*_ is the *i*.*i*.*d*. measurement error. In summary, model A associates weight Z-score to the time periods (EARLY versus LATE), nutrition and medication variables, and their interactions. Model B is much like model A except that it uses microbiota phases to quantify the developmental stages of microbial community instead. For model A, LATE is considered as the baseline phase (coded as 0) and EARLY is coded as 1. For model B, phase 3 is considered as the baseline phase (coded as 0); phases 1 and 2 are coded as 1 in two separate binary variables. The interactions included in both models are defined as the products of the nutrition/medication variables and period/phase-related covariates. The significance of associations is determined by regression *t* test with Satterthwaite’s approximation. Due to the use of large number of covariates in these models, stepwise model selection based on the Akaike information criterion (AIC) was used to reduce model complexity. The results of model B for weight Z-score are summarized in Table 2 of the main text. As an example, the linear associations of P2 and percent lipids * P2 with the weight z-score are both significant (beta = − 0.7766 for P2 and 5.658 for lipids * P2); meaning that while P2 is correlated with a smaller weight z-score as compared with the baseline (P3), a higher percent of lipid intake for P2 subjects increases the weight Z-scores for subjects in P2. Analyses were performed in R 3.2.0 (R Foundation for Statistical Computing, Vienna, Austria).

### Predicting microbiome phases

We performed a mixed-effects logistic regression analyses to study the associations between a host of nutrition- and medication-related covariates and the three microbiota phases on the early and late intervals. We considered P3 as the baseline phase and represented P1 and P2 by two separate binary outcome variables. Gestational age at birth and PMA were included to control for their potential confounding effects. A likelihood ratio test was used to determine the statistical significance of associations. The results are summarized in Tables 3A and B.

## Additional files

Additional file 1: Table S1. Number of significant associations identified in the initial linear mixed-effects regression analysis. Specifically, for each covariate listed in this table, we performed linear mixed-effects regression analyses in which the response variables are microbial taxa abundance and the regressors are as follows: (1) gestational age at birth, (2) post-menstral age, (3) this covariate (main), and (4) the interation between the main covariate and PMA. Regression *t* tests were used to assess the statistical significance of associations. Benjamini-Hochberg multiple testing procedure was used to control false discovery rate at 0.05 level. Listed in this table are the numbers of taxa that are significantly associated with each regressors in these initial regression analyses. (DOCX 17 kb)

Additional file 2: **Figure S1.** Composition bar charts by subject and sampling method. **Figure S2.** Alpha diversity by subject and sampling method observed OTUs. **Figure S3.** Number of Dirichlet components vs. model fit. **Figure S4.** Weighted UniFrac Principal Coordinate Analyses of phase and Dirichlet component. (A) Weighted UniFrac Principal Coordinate Analysis plot colored by phase. (B) Weighted UniFrac Principal Coordinate Analysis plot colored by Dirichlet Component. **Figure S5.** Total functional variance based on the fitted microbiome abundance at the species level relative to postmenstrual age (PMA). **Figure S6.** Linear regression analysis of meconium samples and Phase 1 rectal samples. Comment on Figure 3F. (DOCX 1141 kb)

Additional file 3: Table S2. Confusion table of phases vs. Dirichlet multinomial mixture components. Each sample was classified as representing a specific phase based on the ratios of *Bacilli*, *Gammaproteobacteria*, and *Clostridia*, as described. Independently, each sample was classified as representing a Dirichlet multinomial mixture (DMM) component based on the abundances of all classes of bacteria present. Each row of the table above indicates the number of samples classified as a given phase, and each column indicates the number of samples classified as a given DMM component. Row/column intersections indicate the number of samples classified as the corresponding phase (row) and DMM component (column). DMM components are numbered automatically from the most common to the least common, while phases are numbered according to their order in a model of temporal progression. Phase 1 is equivalent to DMM component 3; phase 2 corresponds to DMM component 2; and phase 3 corresponds to both DMM components 1 and 4. Colors on the row and column labels indicate these correspondence relationships, and colors internal to the table identify sets of samples where the phase-based and DMM component classifications are in agreement. (DOCX 18 kb)

Additional file 4: Table S3. Kruskal-Wallis analysis of variance of all bacterial taxa across the three Phases of the microbiome. (XLSX 109 kb)

Additional file 5: Table S4. A. Results of differential abundance tests (metagenomeSeq zero-inflated Gaussian) of all bacterial taxa between microbiome phases 1 and 2. B. Results of differential abundance tests (metagenomeSeq zero-inflated Gaussian) of all bacterial taxa between microbiome phases 2 and 3. C. Results of differential abundance tests (metagenomeSeq zero-inflated Gaussian) of all bacterial taxa between microbiome phases 1 and 3. (ZIP 536 kb)

Additional file 6: Table S5. Significant results of linear discriminant analysis of putative functional features of microbial communities across the three Phases of the microbiome. (XLSX 24 kb)

Additional file 7: Table S6. Significant associations between bacterial taxa and nutrition, medications, and other clinical factors, within each of the three phases of the microbiome. (XLSX 34 kb)

Additional file 8: Table S7. Multiple regression associations from period-based model with weight Z-score as the outcome variable. The interaction terms are denoted by an asterisk. The *p* values indicate the significance of each association, while the beta values indicate the direction and magnitude of the relationship between weight Z-score and the covariates. (DOCX 12 kb)

Additional file 9: Tables S8. A–B. Full results of mixed-effects logistic regression for nutrition and medication. (DOCX 14 kb)

Additional file 10: Table S9. Linear regression analysis of significant taxa for two variables (genera in P1 and genera in meconium), those that are significant for both variables and those that are unique to each (P1 or meconium). (XLSX 9 kb)

## Abbreviations

BPA: Bisphenol A
DOL: Day of life
LPS: Lipopolysaccharide
NEC: Necrotizing entercolitis
NICU: Neonatal intensive care unit
OTUs: Operational taxonomic units
PMA: Premenstrual age
PROP: Prematurity and Respiratory Outcomes Program
