# Supplementary material for: Impact of prematurity and nutrition on the developing gut microbiome and preterm infant growth
Source: Microbiome. 2017 Dec 11;5:158. doi: 10.1186/s40168-017-0377-0 (PMC5725645; doi:10.1186/s40168-017-0377-0)
Supplement: Supplementary file 1 — Number of significant associations identified in the initial linear mixed-effects regression analysis. Specifically, for each covariate listed in this table, we performed linear mixed-effects regression analyses in which the response variables are microbial taxa abundance and the regressors are as follows: (1) gestational age at birth, (2) post-menstral age, (3) this covariate (main), and (4) the interation between the main covariate and PMA. Regression t tests were used to assess the statistical significance of associations. Benjamini-Hochberg multiple testing procedure was used to control false discovery rate at 0.05 level. Listed in this table are the numbers of taxa that are significantly associated with each regressors in these initial regression analyses. (DOCX 17 kb) [file 40168_2017_377_MOESM1_ESM.docx]

**Additional file 1: Table S1**

| **Covariates** | **Gestational**  **Age at Birth** | **PMA** | **Main** | **Interaction** |
| --- | --- | --- | --- | --- |
| Delivery Method | 0 | 96 | 0 | 2 |
| Antibiotics Past week | 0 | 89 | 60 | 16 |
| Diuretics Past week | 0 | 102 | 27 | 4 |
| Corticosteroid Past Week | 0 | 121 | 9 | 5 |
| Motility Agents Past Week | 0 | 117 | 5 | 13 |
| H_2_ Receptor Antagonists Past Week | 0 | 118 | 6 | 4 |
| Calories/kg Past Week | 0 | 49 | 80 | 3 |
| Ratio of Lipids to Total Calories (g/cal) | 0 | 58 | 78 | 3 |
| Ratio of Proteins to Total Calories (g/cal) | 0 | 56 | 33 | 2 |
| Proportion of Calories Enteral | 0 | 48 | 83 | 16 |

**Number of significant associations identified in the initial linear mixed effects regression analysis.** Specifically, for each covariate listed in this table, we performed linear mixed effects regression analyses in which the response variables are microbial taxa abundance and the regressors are: (1) gestational age at birth; (2) post-menstral age; (3) this covariate (Main); and (4) the interation between the main covariate and PMA. Regression t-tests were used to assess the statistical significance of associations. Benjamini-Hochberg multiple testing procedure was used to control false discovery rate at 0.05 level. Listed in this table are the numbers of taxa that are significantly associated with each regressors in these initial regression analyses.
