# Supplementary material for: Impact of prematurity and nutrition on the developing gut microbiome and preterm infant growth
Source: Microbiome. 2017 Dec 11;5:158. doi: 10.1186/s40168-017-0377-0 (PMC5725645; doi:10.1186/s40168-017-0377-0)
Supplement: Supplementary file 2 — Figure S1. Composition bar charts by subject and sampling method. Figure S2. Alpha diversity by subject and sampling method observed OTUs. Figure S3. Number of Dirichlet components vs. model fit. Figure S4. Weighted UniFrac Principal Coordinate Analyses of phase and Dirichlet component. (A) Weighted UniFrac Principal Coordinate Analysis plot colored by phase. (B) Weighted UniFrac Principal Coordinate Analysis plot colored by Dirichlet Component. Figure S5. Total functional variance based on the fitted microbiome abundance at the species level relative to postmenstrual age (PMA). Figure S6. Linear regression analysis of meconium samples and Phase 1 rectal samples. Comment on Figure 3F. (DOCX 1141 kb) [file 40168_2017_377_MOESM2_ESM.docx]

**Additional file 2**

**
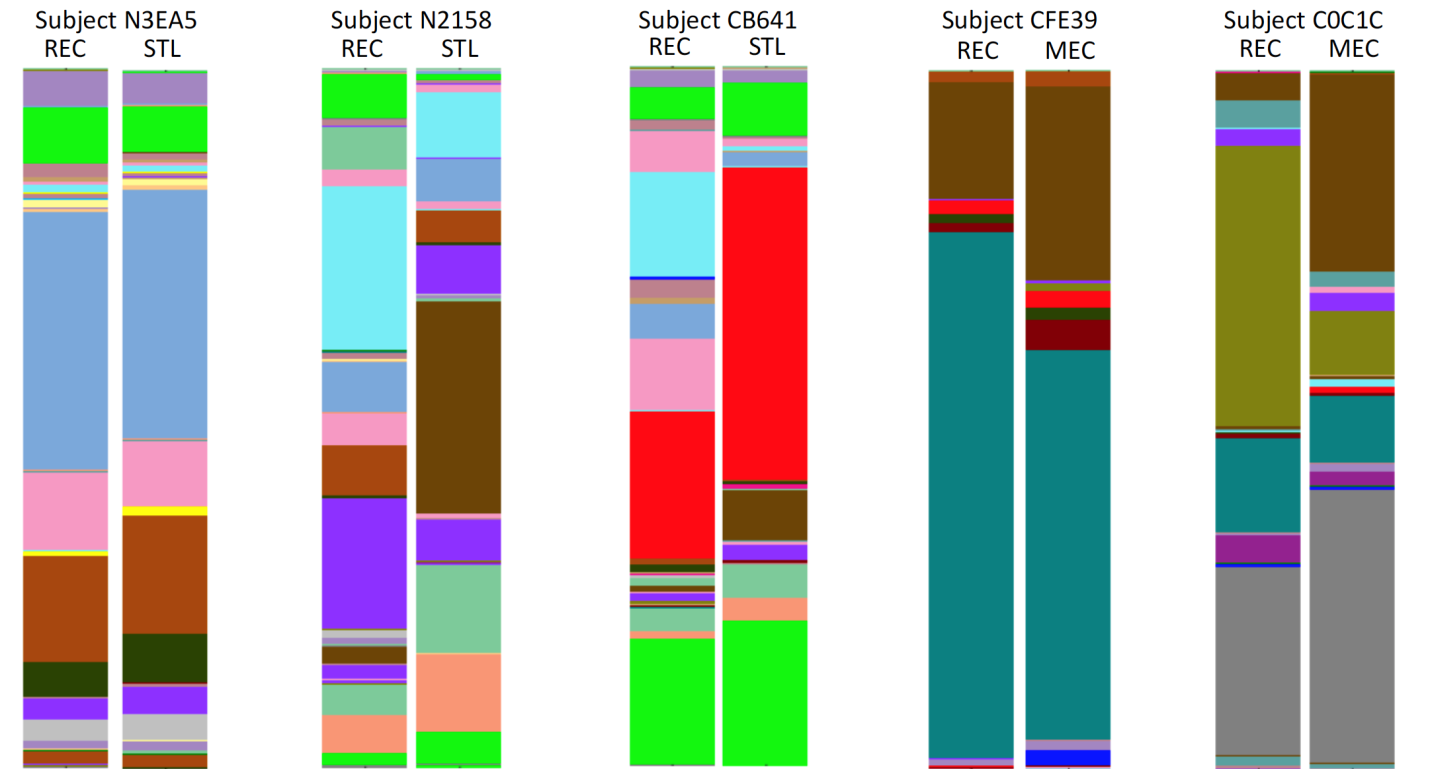
**

**Figure S1. Composition bar charts by subject and sampling method.** Bar charts showing the average composition of matched rectal/meconium (PROP0510203, PROP0510208) or rectal/stool (RPRC0540002, RPRC0530027, RPRC0530031) samples from five preterm infants at the genus level. The composition in rectal/meconium and rectal/stool matched samples is similar in PROP0510203, RPRC0540002, RPRC0530027 and RPRC053003, but diverges in PROP0510208.

**
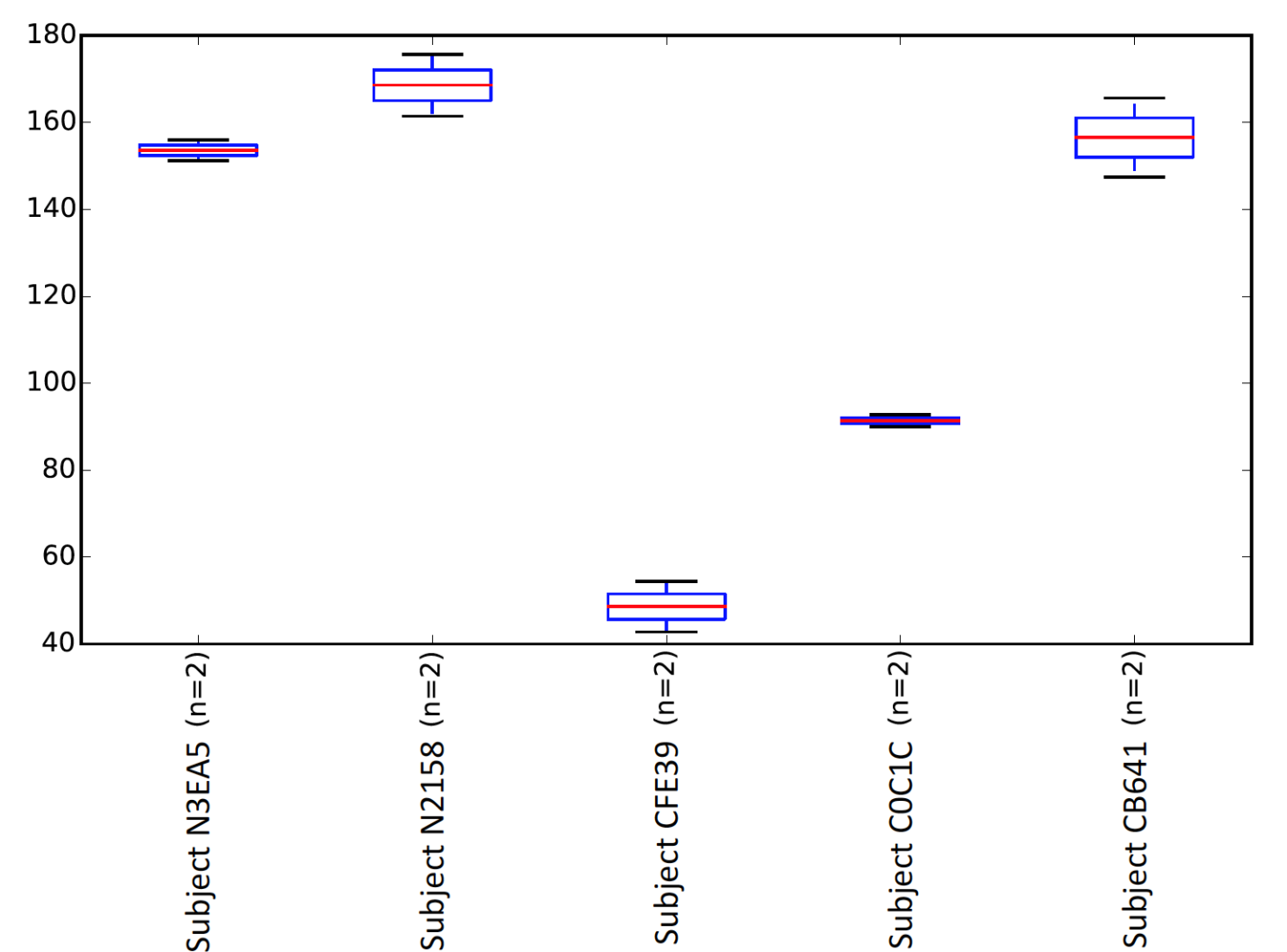
**

**Figure S2. Alpha diversity by subject and sampling method observed OTUs.** Alpha diversity of observed OTUs in matched rectal/meconium (PROP0510203, PROP0510208) or rectal/stool (RPRC0530027, RPRC0530031, RPRC0540002) samples. Both sampling methods capture a similar alpha diversity or evenness of observed OTUs within a subject and greater diversity between subjects.

**Figure S3.** **Number of Dirichlet components vs. model fit.**

Dirichlet Multinomial mixture (DMM) models were fit for the class level composition of all samples in an iterative fashion, incorporating one through ten Dirichlet components. The model fit was estimated using the Laplace approximation of model evidence for each number of components, where a lower value indicates a better model fit. Accordingly, the model using four Dirichlet components was selected.


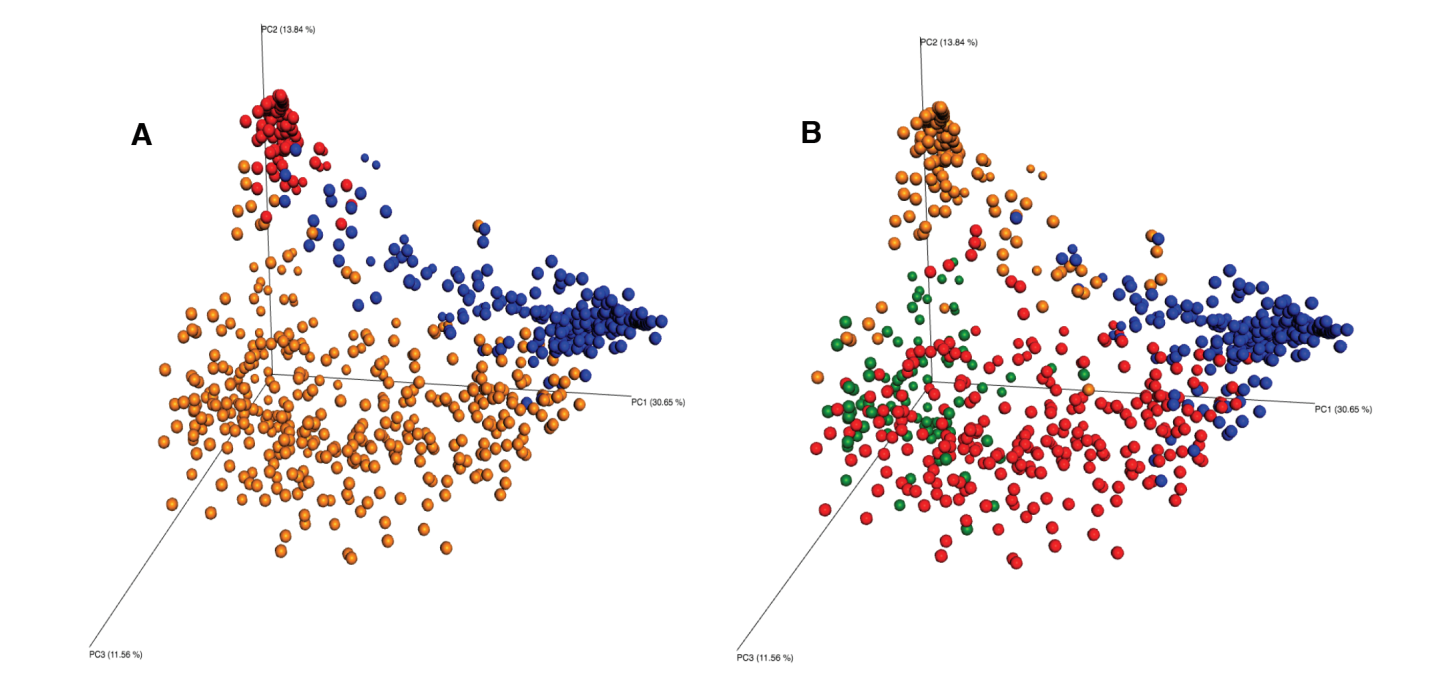


**Figure S4. Weighted UniFrac Principal Coordinate Analyses of phase and Dirichlet component. (A)** **Weighted UniFrac Principal Coordinate Analysis plot colored by phase.** Weighted UniFrac Distances were calculated between all pairs of samples after rarefying to an even depth of 12,000 reads. These distances were used to perform principal coordinate analysis, the first three axes of which were used to construct the adjacent plot. Samples are colored according to their phase classification (Phase 1, red [90 samples]; Phase 2, blue [244 samples]; Phase 3, tan [387 samples]). The overall structure of the samples in the plot resembles a triangle, with phase one samples clustered at the top, phase two samples clustered at the lower right, and phase three samples clustered at the lower left. **(B) Weighted UniFrac Principal Coordinate Analysis plot colored by Dirichlet Component.** Weighted UniFrac Distances were calculated between all pairs of samples after rarefying to an even depth of 12,000 reads. These distances were used to perform principal coordinate analysis, the first three axes of which were used to construct the adjacent plot. Samples are colored according to their Dirichlet component classification (DMM1, red [262 samples]; DMM2, blue [234 samples]; DMM3, tan [132 samples]; DMM4, green [93 samples]). The overall structure of the samples in the plot resembles a triangle, with component one samples in the lower middle, component two samples in the lower right, component three samples at the top, and component four samples at the lower left. Components three, two, and four correspond to phases one, two, and three, respectively. Component one appears to represent an intermediate stage between phases two and three. However, of the 262 component one samples, 254 are classified as phase three and only eight are classified as phase two, indicating that component one corresponds to a less mature sub-type of phase three.


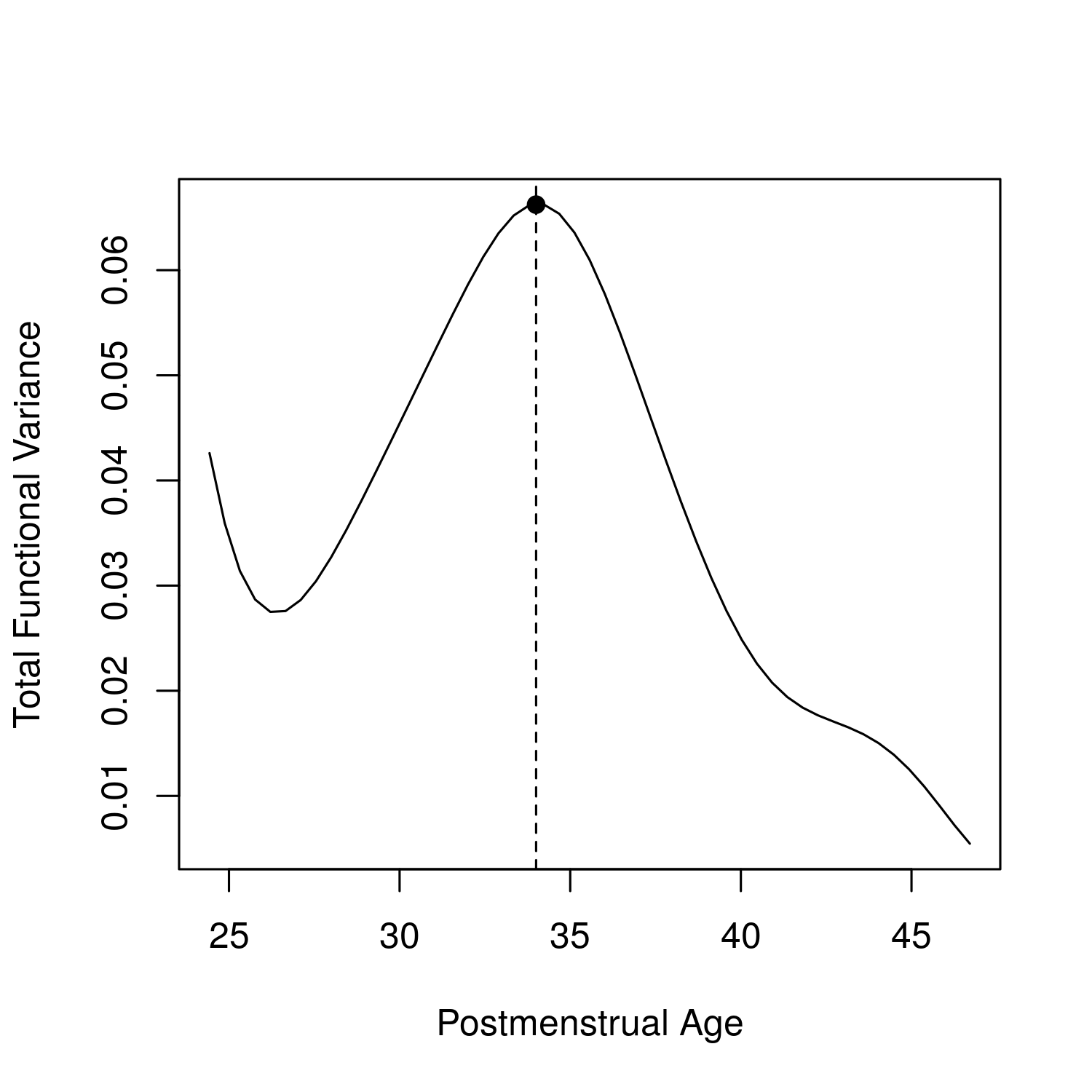


**Figure S5. Total functional variance based on the fitted microbiome abundance at the species level relative to postmenstrual age (PMA).** Maximum total functional variance occurred at PMA = 34 weeks. Based on this peak variance, the EARLY period of PMA was defined as < 34 weeks; n=362 data points. The LATE period of PMA was defined as ≥ 34 weeks; n=343 data points. The calculation of total function variance is described in Online Methods.

**A**

| **Samples** | Meconium | Not Meconium | Total |
| --- | --- | --- | --- |
| Phase 1 | 58 | 32 | 90 |
| Not Phase 1 | 39 | 592 | 631 |
| Total | 97 | 624 | 721 |
| **B** |  |  |  |
|  |  |  |  |
| **Significant Associations (Genus Level)** | Meconium | Not Meconium | Total |
| Phase 1 | 34 | 6 | 40 |
| Not Phase 1 | 3 | 313 | 316 |
| Total | 37 | 319 | 356 |

**Figure S6. Linear regression analysis of meconium samples and Phase 1 rectal samples. (A)** From the 721 preterm samples included in the longitudinal analysis, samples were identified as meconium if the infant had not transitioned to normal stool. Phase 1 samples were identified according to the decision tree. All meconium samples are not exclusively in Phase 1. **(B)** Number of genera significantly associated with either meconium or Phase 1. The total number of genera associated with meconium is nearly identical to genera associated with Phase 1.

**Comment on Figure 3F.** We note that Stewart et al cultured the unclassified *Enterobacteriaceae* from preterm infant fecal samples in their study and classified it as *Klebsiella* using MALDI-TOF-MS and full-length 16S rRNA sequencing [1]. In our analysis, we identified the *Klebsiella* genus as distinct from the unclassified *Enterobacteriaceae*. Without additional full-length 16S rRNA discrimination of unclassified *Enterobacteriaceae* from our samples, we refer to them as unclassified *Enterbacteriaceae*.

**Reference**

1. Stewart CJ, Embleton ND, Marrs EC, Smith DP, Nelson A, Abdulkadir B, Skeath T, Petrosino JF, Perry JD, Berrington JE *et al*: **Temporal bacterial and metabolic development of the preterm gut reveals specific signatures in health and disease**. *Microbiome* 2016, **4**(1):67.
