# Supplementary material for: Impact of prematurity and nutrition on the developing gut microbiome and preterm infant growth
Source: Microbiome. 2017 Dec 11;5:158. doi: 10.1186/s40168-017-0377-0 (PMC5725645; doi:10.1186/s40168-017-0377-0)
Supplement: Supplementary file 3 — Confusion table of phases vs. Dirichlet multinomial mixture components. Each sample was classified as representing a specific phase based on the ratios of Bacilli, Gammaproteobacteria, and Clostridia, as described. Independently, each sample was classified as representing a Dirichlet multinomial mixture (DMM) component based on the abundances of all classes of bacteria present. Each row of the table above indicates the number of samples classified as a given phase, and each column indicates the number of samples classified as a given DMM component. Row/column intersections indicate the number of samples classified as the corresponding phase (row) and DMM component (column). DMM components are numbered automatically from the most common to the least common, while phases are numbered according to their order in a model of temporal progression. Phase 1 is equivalent to DMM component 3; phase 2 corresponds to DMM component 2; and phase 3 corresponds to both DMM components 1 and 4. Colors on the row and column labels indicate these correspondence relationships, and colors internal to the table identify sets of samples where the phase-based and DMM component classifications are in agreement. (DOCX 18 kb) [file 40168_2017_377_MOESM3_ESM.docx]

| Phase\DMM Component | C1 | C2 | C3 | C4 |
| --- | --- | --- | --- | --- |
| P1 | 0 | 0 | 90 | 0 |
| P2 | 8 | 211 | 25 | 0 |
| P3 | 254 | 23 | 17 | 93 |

**Additional file 3: Table S2.** **Confusion table of Phases vs. Dirichlet multinomial mixture components.** Each sample was classified as representing a specific phase based on the ratios of *Bacilli*, *Gammaproteobacteria*, and *Clostridia*, as described. Independently, each sample was classified as representing a Dirichlet multinomial mixture (DMM) component based on the abundances of all classes of bacteria present. Each row of the table above indicates the number of samples classified as a given phase, and each column indicates the number of samples classified as a given DMM component. Row/column intersections indicate the number of samples classified as the corresponding phase (row) and DMM component (column). DMM components are numbered automatically from most common to least common, while phases are numbered according to their order in a model of temporal progression. Phase 1 is equivalent to DMM component 3; Phase 2 corresponds to DMM component 2; and Phase 3 corresponds to both DMM components 1 and 4. Colors on the row and column labels indicate these correspondence relationships, and colors internal to the table identify sets of samples where the phase-based and DMM component classifications are in agreement.

These observations prompted us to examine why the DMM model split our phase three samples into two DMM components (components one and four). Upon inspection, DMM component four appears to have more of the canonical characteristics of phase three (mean *Clostridia* abundance = 48.5%, *Gammaproteobacteria* = 9.3%, Bacilli = 12.8%), while DMM component one appears to be comprised of samples that exhibit characteristics of both phases two and three, with phase three characteristics being prominent (mean *Clostridia* abundance = 39.9%, *Gammaproteobacteria* = 36.8%, *Bacilli* = 14.2%). These findings suggest that DMM component one may represent a sort of intermediate or transitional period during which phase three features manifest while phase two features persist in a limited way. These relationships between phases and DMM components are evident in Weighted UniFrac PCoA plots with samples colored according to phase and DMM component **(Supplemental Material, Figures S4A-B)**. Samples are concentrated at three corners of a triangle-like structure with each corner corresponding to a phase. Coloring the samples by DMM component reveals that component one samples are concentrated in the region between the phase two and phase three clusters. The temporal distribution of DMM components one and four are consistent with these observations, with the mean day of life and the mean post menstrual age of component four samples being approximately two weeks later than those of component one samples (p-value ≈ 0.0).

We believe the results of this DMM model-based analysis support our phase-based clustering approach, as evidenced by the high degree of concordance between phase and DMM component classification. The fact that the best DMM model fit was achieved with four components instead of three, and that this corresponds to splitting our phase three cluster into two sub-clusters, is not inconsistent or incompatible with our phase-based paradigm. In the phase model, the *Bacilli* characteristic of phase one give way to the *Gammaproteobacteria* characteristic of phase two and diminish dramatically – often nearly disappearing – in the process. The transition between phase two and three differs in that phase three is not distinguished so much by the disappearance of *Gammaproteobacteria* as it is by the sudden emergence of *Clostridia* at significant levels. *Gammaproteobacteria* are seen to persist at appreciable abundances in phase three, while *Clostridia* appear to bloom and then continue to flourish. Accordingly, the primary distinction between DMM components two and one is the prominence of *Clostridia* in component one. The distinction between DMM components one and four is less of a bright line, and is characterized by substantial abundance of *Clostridia* concurrent with significantly diminished *Gammaproteobacteria* in component four. The hallmarks of phase three – significant abundance of *Clostridia* and high diversity – are manifest in both DMM components one and four, with component four representing a more mature sub-type of phase three. Both components one and four are easily distinguishable from phases one and two, and both likely indicate the developmental milestone that we have identified as phase three, which is characterized by the ability of the infant gut to support substantial levels and varieties of obligate anaerobic *Clostridia*.
