# Supplementary material for: Impact of prematurity and nutrition on the developing gut microbiome and preterm infant growth
Source: Microbiome. 2017 Dec 11;5:158. doi: 10.1186/s40168-017-0377-0 (PMC5725645; doi:10.1186/s40168-017-0377-0)
Supplement: Supplementary file 8 — Multiple regression associations from period-based model with weight Z-score as the outcome variable. The interaction terms are denoted by an asterisk. The p values indicate the significance of each association, while the beta values indicate the direction and magnitude of the relationship between weight Z-score and the covariates. (DOCX 12 kb) [file 40168_2017_377_MOESM8_ESM.docx]

**Additional file 8: Table S7**

| **Covariates** | ***p*-value** | **Beta value** |
| --- | --- | --- |
| Gestational Age at Birth | 0.0061 | 0.06595 |
| Calories/kg Past Week | < 0.0001 | -0.7713 |
| Ratio of Lipids to Total Calories (g/cal) | 0.0004 | -8.81 |
| Antibiotics Past Week | < 0.0001 | -0.3668 |
| Diuretics Past Week | < 0.0001 | -0.2568 |
| Corticosteroids Past Week | 0.0369 | 0.4465 |
| Motility Agents Past Week | 0.0469 | 0.101 |
| Calories/kg Past Week * Early Period | 0.0138 | 0.267 |
| Proportion of Calories Enteral * Early Period | 0.0008 | -0.3163 |
| Antibiotics Past Week * Early Period | < 0.0001 | 0.2702 |
| Diuretics Past Week * Early Period | < 0.0001 | 0.2821 |
| Corticosteroids Past Week * Early Period | 0.0356 | -0.4593 |

**Multiple regression associations from period based model with weight Z-score as the outcome variable.** The interaction terms are denoted by an asterisk. The p-values indicate the significance of each association, while the beta values indicate the direction and magnitude of the relationship between weight Z-score and the covariates.
