# Supplementary material for: Impact of prematurity and nutrition on the developing gut microbiome and preterm infant growth
Source: Microbiome. 2017 Dec 11;5:158. doi: 10.1186/s40168-017-0377-0 (PMC5725645; doi:10.1186/s40168-017-0377-0)
Supplement: Supplementary file 9 — A–B. Full results of mixed-effects logistic regression for nutrition and medication. (DOCX 14 kb) [file 40168_2017_377_MOESM9_ESM.docx]

**Additional file 9: Table S8. A-B. Full results of mixed effects logistic regression for nutrition and medication.**

| **Covariates** | **Phase 1**  **p-value** | **Phase 2**  **p-value** | **Phase 1**  **Beta value** | **Phase 2**  **Beta value** |
| --- | --- | --- | --- | --- |
| Gestational Age At Birth | 0.6251 | 0.1354 | 0.0965 | -0.1811 |
| PMA (week) | 0.032 | 0.1674 | -0.3831 | 0.1403 |
| Calories/Kg Past Week | 0.3042 | 0.0663 | -2.598 | 2.601 |
| Ratio of Lipids to Total Calories (g/cal) | 0.1913 | 0.0002 | -57.74 | 119.8 |
| Ratio of Proteins to Total Calories (g/cal) | 0.0092 | 0.0053 | 110.1 | -70.12 |
| Proportion of Calories Enteral | 0.8786 | <0.0001 | -0.2702 | -6.039 |
| Antibiotics Past Week | 0.5738 | 0.0778 | 0.3194 | 0.582 |
| Diuretics Past Week | 0.007 | 0.1053 | -1.457 | 0.5927 |
| Corticosteroids Past Week | 0.9608 | 0.823 | -0.037 | 0.0980 |
| Motility Agents Past Week | 0.999 | 0.5287 | -19.23 | -0.3484 |

**Table S8A.** Results of mixed effects logistic regression analysis between nutrition/medication and microbiome phases during the EARLY period (<34 weeks PMA). Phase 1 and Phase 2 are considered as binary outcome variables (Yes/No) and are analyzed separately. Beta values are the estimated regression coefficients and p-values are computed from the likelihood-ratio tests.

| **Covariates** | **Phase 1**  **p-val** | **Phase 2**  **p-val** | **Phase 1 Beta value** | **Phase 2 Beta value** |
| --- | --- | --- | --- | --- |
| Gestational Age At Birth | 0.9618 | 0.0608 | 1.385 | -0.2558 |
| PMA (week) | 0.3467 | 0.0011 | -7.108 | -0.4875 |
| Calories/Kg Past Week | 0.9513 | 0.8137 | -11.09 | 0.5573 |
| Ratio of Lipids to Total Calories (g/cal) | 0.8278 | 0.1076 | -917.5 | 93.3 |
| Ratio of Proteins to Total Calories (g/cal) | 0.9455 | 0.0259 | -84.96 | -98.05 |
| Proportion of Calories Enteral | 0.7124 | 0.0017 | 15.29 | -6.428 |
| Antibiotics Past Week | 0.8903 | 0.0014 | 9.91 | 1.903 |
| Diuretics Past Week | 0.5951 | 0.1722 | -20.98 | -0.951 |
| Proton Pump Inhibitors Past Week | 1 | 0.3001 | 8.971 | 1.46 |
| H2 Receptor Antagonists Past Week | 0.9936 | 0.3006 | 7.75 | 1.313 |
| Motility Agents Past Week | 0.8556 | 0.2154 | 7.116 | -0.9349 |

**Table S8B.** Results of mixed effects logistic regression analysis between nutrition/medication and microbiome phases during the LATE period (≥34 weeks PMA). Phase 1 and Phase 2 are considered as binary outcome variables (Yes/No) and are analyzed separately. Beta values are the estimated regression coefficients and p-values are computed from the likelihood-ratio tests.
